# The impact of COVID-19 during pregnancy on maternal and neonatal outcomes: a systematic review

**DOI:** 10.14806/ej.26.1.969

**Published:** 2021-10-22

**Authors:** Despoina Michailidou, Androniki Stavridou, Eleni D. Panagouli, Theodoros N. Sergentanis, Theodora Psaltopoulou, Flora Bacopoulou, Valentina Baltag, Donald E. Greydanus, George Mastorakos, George P. Chrousos, Maria N. Tsolia, Artemis K. Tsitsika, Nikolaos Vlahos

**Affiliations:** 1 Adolescent Health Unit, Second Department of Pediatrics, “P. & A. Kyriakou” Children’s Hospital, National and Kapodistrian University of Athens, Athens, Greece; 2 Department of Clinical Therapeutics, School of Medicine, National and Kapodistrian University of Athens, Athens, Greece; 3 University Research Institute of Maternal and Child Health & Precision Medicine and UNESCO Chair on Adolescent Health Care, National and Kapodistrian University of Athens, Aghia Sophia Children’s Hospital, Athens, Greece; 4 Department of Maternal, Newborn, Child & Adolescent Health & Ageing, World Health Organization; 5 Department of Pediatric and Adolescent Medicine, Western Michigan University Homer Stryker M.D. School of Medicine, United States; 6 Second Department of Obstetrics and Gynecology, National and Kapodistrian University of Athens, School of Medicine, Aretaieion Hospital, Athens, Greece

## Abstract

Several months after the onset of the epidemic, COVID-19 remains a global health issue. Scientific data on pregnancy, perinatal outcomes and vertical transmission of SARS-CoV-2 are constantly emerging but are still limited and unclear. The purpose of this systematic review was to summarize current evidence on vertical transmission rates, maternal, perinatal and neonatal outcomes and mode of delivery in pregnancies affected by COVID-19. An extensive search was conducted in PubMed, Google Scholar, Embase, and Scopus databases up to June 20, 2020. A total of 133 articles (51 case reports, 31 case series, 40 cohort studies and 2 case-control studies) reporting data from 8,092 subjects (6,046 pregnant women and 2,046 neonates) were considered eligible for inclusion in the systematic review. A substantial proportion of pregnant women with COVID-19 underwent caesarean section (case reports 82.2%, case series 74.2% and cohort studies 66.0%). Regarding vertical transmission, most neonates were tested negative (case reports 92.7%, case series studies 84.2%, cohort studies 97.1% and case control studies 100%). Maternal mortality rates ranged from 1% in cohort studies to 5.7% in case reports; neonatal mortality ranged from 2% in case reports to 3.3% in case series. Vertical transmission of SARS-CoV-2 from mother to child is rare. Careful screening of pregnant women seems important and specific guidelines with evidence-based decision algorithms for the mode of delivery in the context of a pregnancy affected by COVID-19 should be established.

## Introduction

Coronavirus disease (COVID-19) is caused by SARS-CoV-2 (severe acute respiratory syndrome coronavirus 2) and was first described in Wuhan, China in December of 2019 (Jin et al., 2020). COVID-19 was declared by the World Health Organization as pandemic on March 11, 2020 (Ng et al., 2020) and can result in severe pneumonia, multi-organ failure and death (Hui et al., 2020). Over the last twenty years, two large epidemics of coronaviruses have been recorded, the SARS (Severe Acute Respiratory Syndrome) epidemic with a case fatality rate about 10.5% (WHO) and the MERS (Middle East Respiratory Syndrome) epidemic with a case fatality rate of 34.3% (WHO). According to the literature, the infections caused from SARS and MERS coronaviruses have been associated with serious maternal and neonatal morbidity and mortality, stillbirth and high percentage of spontaneous abortion (Schwartz and Graham, 2020; Wong et al., 2004). The epidemiological data from China about COVID-19 have shown that most cases had mild symptoms with a case fatality rate about 2.3%. SARS-CoV-2 seems to be more contagious on close contacts (Wu and McGoogan, 2019), albeit less aggressive than the aforementioned two coronaviruses.

Pregnancy is considered a state of relative immunological suppression, with a reduction in cellular immunity and potential susceptibility to infections (Birkeland and Kristoffersen, 1980; Goodnight and Soper, 2005); changes in hormonal levels, such as beta human chorionic gonadotropin (β-hCG), progesterone and cortisol may mediate pregnancy-related cellular immunity immunosuppression. Additionally, the increase in uterine size causes the diaphragm to rise by 4 cm, enlarging the transverse diameter of the chest by 2 cm and affecting pulmonary volume. On the other hand, the immaturity of the immune system of fetuses and neonates makes them more vulnerable to infections (van Well et al., 2017). Therefore, pregnant women and neonates could be considered a high-risk group for infection during the present pandemic.

Scientific data on pregnancy, perinatal outcomes and vertical transmission of SARS-CoV-2 are rather limited but rapidly accumulating. Over the past months, case reports and cohort studies have reported variable results on the mode of delivery, perinatal outcomes, vertical transmission from mother to infant or intrauterine transmission, and treatment modalities (Lang and Zhao, 2020; Martinelli et al., 2020; Ahmed et al., 2020; Liu W et al., 2020). The purpose of this systematic review was to summarize current evidence on vertical transmission rates, maternal, perinatal and neonatal outcomes and mode of delivery in pregnancies affected by COVID-19.

## Materials, Methodologies and Techniques

### Study design

The present systematic review was performed according to the PRISMA guidelines (Liberati et al., 2009). A search was performed in PubMed, Google Scholar, Embase, and Scopus databases up to June 20, 2020. The following search terms were used: (Covid-19 OR COVID-19 OR SARS-CoV-2 OR “2019-nCoV” OR “novel coronavirus”) AND (gestation OR pregnancy OR pregnant OR gestational OR neonate OR neonatal). Additionally, references of all articles were checked thoroughly.

#### Inclusion criteria

Only original research articles (cohort studies, cross-sectional studies, case-control studies, case series and case reports) published in the English language were included. Studies referring to pregnancies and/or deliveries of all ages with maternal confirmed COVID-19, with reverse transcription polymerase chain reaction (RT-PCR) and/or positive computed tomography (CT) findings, were deemed eligible. No limitations, such as ethnicity or journal, were considered. Studies with overlapping populations were excluded.

### Eligibility assessment and risk of bias assessment

The retrieved studies were screened by three reviewers independently (D.M., A.S. and E.P.). The Newcastle-Ottawa Quality Scale (Ottawa Hospital Research Institute) was used to evaluate the quality of the studies. If there was a disagreement, team consensus followed.

### Data extraction

Three authors (D.M, A.S. and E.P.) extracted all relevant data. General information, such as first author’s name, location or country, study design, study period, number of participants (pregnancies or neonates), maternal age, gestational age, mode of delivery, treatment, co-morbidity of the mother, maternal and neonatal outcomes and test for COVID-19 were recorded. Data were tabulated; frequencies and percentages for categorical variables were estimated.

## Results

### Selection of studies

**Figure 1.**
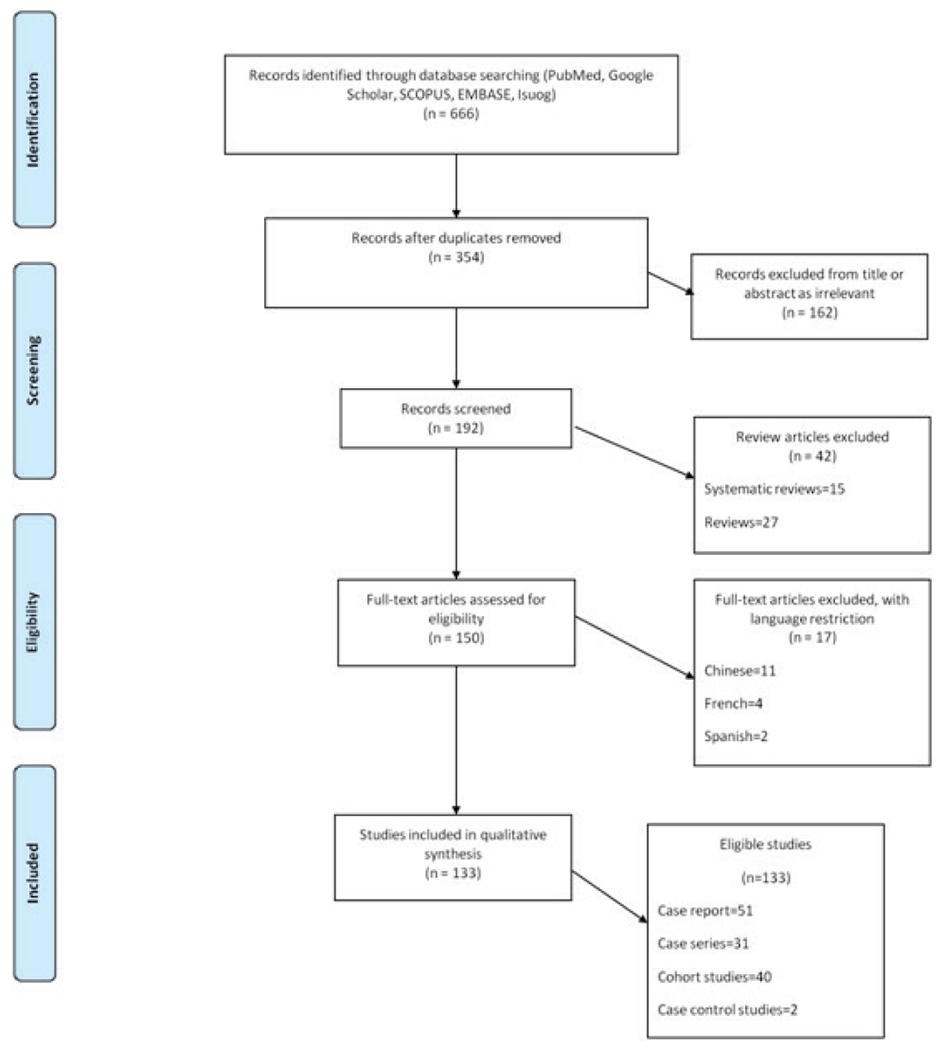
PRISMA flow chart of included studies.

After search in the databases, a total of 666 articles were retrieved. The flow chart describing the selection of studies is presented in Figure 1.

After removal of duplicates, 354 items were selected for extensive review. Out of them, 162 articles were excluded as irrelevant to the topic and 42 articles as reviews (15 systematic reviews and 27 reviews). The remaining 150 full-text articles were subjected to further consideration; of them, 17 articles were excluded because of language (eleven in Chinese, four in French, and two in Spanish). Finally, 133 articles were included in the systematic review (Wang S et al., 2020; Dong L et al., 2020; Wen et al., 2020; Lee et al., 2020; Kalafat et al., 2020; Chen R, Chen et al., 2020; Peng et al., 2020; Lowe and Bopp, 2020; Xiong et al., 2020; Wang et al., 2020; Li et al., 2020; Lang and Zhao, 2020; Schnettler et al., 2020; Iqbal et al., 2020; Piersigilli et al., 2020; Taghizadieh et al., 2020; Xia et al., 2020; Lu et al., 2020; Hong et al., 2020; Blauvelt et al., 2020; Lyra et al., 2020; Kelly et al., 2020; Martinelli et al., 2020; Du et al., 2020; Bani et al., 2020; Rabice et al., 2020; AlZaghal et al., 2020; Nesr et al., 2020; Carosso et al., 2020; Yu et al., 2020; Oh et al., 2020; Kuhrt et al., 2020; Ahmed et al., 2020; Kirtsman et al., 2020; Mehta et al., 2020; Anderson et al., 2020; Dong Y et al., 2020; De Socio et al., 2020; Yilmaz et al., 2020; Baud et al., 2020; Fontanella et al., 2020; Gidlöf et al., 2020; Alonso Díaz et al., 2020; Liao X et al., 2020; Mohammadi et al., 2020; Sinkey et al., 2020; Panichaya et al., 2020; Zamaniyan et al., 2020; Zambrano et al., 2020; Liao et al., 2020; Liu et al., 2020; Yu et al., 2020; Chen et al., 2020; Zhu et al., 2020; Fan et al., 2020; Zeng et al., 2020; Yang et al., 2020; Chen et al., 2020; Vlachodimitropoulou et al., 2020; Khan et al., 2020; Buonsenso et al., 2020; Wu C et al., 2020; Hu et al., 2020; Hantoushzadeh et al., 2020; Baergen and Heller, 2020; Buonsenso et al., 2020; Yu et al., 2020; Juusela et al., 2020; Silverstein et al., 2020; Yassa et al., 2020a; Lucarelli et al., 2020; Govind et al., 2020; Cooke et al., 2020; Dória et al., 2020; Xu et al., 2020; Cao et al., 2020; Andrikopoulou et al., 2020; Huang et al., 2020; McLaren et al., 2020; Breslin et al., 2020; Chen R, Zhang, et al., 2020; Liu H et al., 2020; Liu et al., 2020; Yan et al., 2020; Wu Y et al., 2020; Shanes et al., 2020; Ferrazzi et al., 2020; Penfield et al., 2020; Collin et al., 2020; Qadri and Mariona, 2020; Qiancheng et al., 2020; Wu Xiaoqing et al., 2020; Pierce-Williams et al., 2020; Yang H et al., 2020; Kayem et al., 2020; Knight et al., 2020; Khalil et al., 2020; Chen L, Li, et al., 2020; Lokken et al., 2020; Liao J et al., 2020; London et al., 2020; Patané et al., 2020; Savasi et al., 2020; Pereira et al., 2020; Khoury et al., 2020; Zeng Y et al., 2020; Sentilhes et al., 2020; Zeng Q-L et al., 2020; Mendoza et al., 2020; Wang Z et al., 2020; Martínez-Perez et al., 2020; Miller et al., 2020; Blitz et al., 2020; Fox and Melka, 2020; Campbell et al., 2020; Emeruwa et al., 2020; Gagliardi et al., 2020; Goldfarb et al., 2020; Li N et al., 2020; Tekbali et al., 2020; Sutton et al., 2020; Fassett et al., 2020; RCOG; Ashokka et al., 2020; Zaigham and Andersson, 2020; Juan et al., 2020; Kotabagi et al., 2020; Ifdil et al., 2020; Zeng L-N et al., 2020; Masjoudi et al., 2020; Wu Yanting et al., 2020; Lee T-Y et al., 2020; Suzuki 2020; Huang J-W et al., 2020; Berthelot et al., 2020; Durankuş and Aksu, 2020). Among them 51 were case reports (100 patients, 52 pregnant women and 48 neonates), 31 case series (390 patients, 211 pregnant women and 179 neonates), 40 cohort studies (4,474 patients, 2,685 pregnant women and 1,789 neonates) and 2 case-control studies (3,128 patients, 3,098 pregnant women and 30 neonates). A total of 8,092 patients, 6,046 pregnant women and 2,046 neonates were included in the systematic review.

### Case reports

#### Demographics in case reports:

The 51 case reports (Wang S et al., 2020; Dong L et al., 2020; Alzamora et al., 2020; Wen et al., 2020; Lee DH et al., 2020; Kalafat et al., 2020; Chen R., Chen, et al., 2020; Li Y et al., 2020; Peng et al., 2020; Lowe and Bopp, 2020; Xiong et al., 2020; Wang X et al., 2020; Li J et al., 2020; Lang and Zhao, 2020; Schnettler et al., 2020; Iqbal et al., 2020; Piersigilli et al., 2020; Taghizadieh et al., 2020; Xia et al., 2020; Lu et al., 2020; Hong et al., 2020; Blauvelt et al., 2020; Lyra et al., 2020; Kelly et al., 2020; Martinelli et al., 2020; Du et al., 2020; Bani Hani et al., 2020; Rabice et al., 2020; AlZaghal et al., 2020; Nesr et al., 2020; Carosso et al., 2020; Yu Y et al., 2020; Oh et al., 2020; Kuhrt et al., 2020; Ahmed et al., 2020; Kirtsman et al., 2020; Mehta et al., 2020; Anderson et al., 2020; DongY. et al., 2020; De Socio et al., 2020; Yilmaz et al., 2020; Baud et al., 2020; Fontanella et al., 2020; Gidlöf et al., 2020; Alonso Díaz et al., 2020; Liao X et al., 2020; Mohammadi et al., 2020; Sinkey et al., 2020; Panichaya et al., 2020; Zamaniyan et al., 2020; Zambrano et al., 2020) identified 52 pregnant women (mean ± SD; age 29.7 ± 9.1 years; gestational age 33.1 ± 6.3 weeks) from January 2020 to May 2020. The characteristics of case reports are summarized in Table 1 in Supplementary Data^1^. The cases derived mostly from China (n=15), with US being the second most frequent location (n=11), followed by Italy, UK, Iran, Jordan, and Turkey (n=3), while Korea, Honduras, Sweden, Peru, Australia, Thailand, Spain, Switzerland, Portugal, Belgium, contributed one case each. All women presented to the emergency room for respiratory complications. Among them, 29 women had co-morbid health conditions, *i.e.* gestational diabetes (n=9), obesity (n=9), hypothyroidism (n=5), hypertension (n=3), asthma (n=2), thalassemia (n=1), myotonic dystrophy (n=1), pneumonia (n=1), respiratory failure (n=1), HELLP (hemolysis, elevated liver enzymes, and low platelet count) syndrome (n=1), hepatitis B (n=1), thyroidectomy (n=1), immune thrombocytopenia (ITP) (n=1) and cholecystitis (n=1). All 52 pregnant women had COVID-19, confirmed either through RT-PCR or RT-PCR and CT scans.

#### Data about delivery in case reports:

From the 52 pregnant women, 45 delivered (86%, 45/52) with caesarean section (C-section) (82.2%, 37/45); 7 delivered (15.6%, 7/45) by vaginal route. In one case the mode of delivery was not reported (2.2%, 1/45). From the remaining cases, one had a miscarriage (2%, 1/52), one pregnancy was terminated due to fetal Down syndrome (2%, 1/52) and in 5 cases pregnancy was still on-going or delivery was not reported (10%, 5/52). Fetal distress (19.5%, 7/37), respiratory distress/dyspnea of the mother and pneumonia due to COVID-19 (16.7%, 6/37) and preeclampsia (5.6%, 2/37) were the most common indications for C-section delivery (Table 1 in Supplementary Data^1^). In 12 cases the reason for C-section was not reported (12/37, 37.5%).

#### Therapeutic management in case reports:

Thirty-seven (71.1%, 37/52) women received medication, including hydroxychloroquine (27%, 10/37), azithromycin (27%, 10/37), oseltamivir (16.2%, 6/37), ceftriaxone (13.5%, 5/37), O2 support (13.5%, 5/37), lopinavir (8.1%, 3/37), corticosteroids (without information about the specific agent administered) (13.5%, 5/37), methylprednisolone (8.1%, 3/37), dexamethasone (5.4%, 2/37), remdesivir (5.4%, 2/37), interferon (5.4%, 2/37), oxytocin (2.7%, 1/37) and plasma (2.7%, 1/37). Administration of antivirals (18.9%, 7/37) and antibiotics (21.6%, 8/37) without any other specification were also reported. Due to COVID-19 complications 13 pregnant women were admitted to intensive care unit (ICU) (25% admission rate, 13/52) (Table 1 in Supplementary Data^1^).

#### Neonatal and maternal outcomes in case reports:

A total of 48 neonates were born from the 45 deliveries, including 3 pairs of twins (12.5%, 6/48). The majority of neonates were tested negative for SARS-CoV-2 (84.3%, 32/38), 6 (15.7%, 6/38) were positive for the virus, while for 10 neonates it was not mentioned whether they were tested or not (20.9%, 10/48). Thirty-two neonates were considered healthy (66.6%, 32/48), while 10 were admitted to neonatal intensive care unit (NICU) (20.8%, 10/48) due to prematurity (20%, 2/10), feeding difficulties (10%, 1/10) and precautionary measures for COVID-19 (70%, 7/10). One death was reported 2 hours after birth (2%, 1/48) probably due to maternal COVID-19 and rapid deterioration of her health.

Concerning mother’s health, 10 pregnant women (19.2%, 10/52) were in good condition after hospitalization, 13 were discharged (25%, 13/52), 3 were still hospitalized (5.7%, 3/52) and 3 (5.7%, 3/52) succumbed to COVID-19 complications (Table 1 in Supplementary Data^1^).

### Case series

#### Demographics in case series:

From the 31 case series, 16 derived from China, 5 from New York City, 2 from Italy, 2 from the UK, 2 from New Jersey, and 1 from Canada, Iran, Portugal and Turkey, respectively, as presented in Table 2 in Supplementary Data^1^ (Chen S , et al., 2020; Liu Y et al., 2020; Yu N et al., 2020; Chen H et al., 2020; Zhu et al., 2020; Fan et al., 2020; Zeng L et al., 2020; Yang P et al., 2020; Chen Y., Peng, et al., 2020; Vlachodimitropoulou Koumoutsea et al., 2020; Khan et al., 2020; Buonsenso et al., 2020; Wu C et al., 2020; Hu et al., 2020; Hantoushzadeh et al., 2020; Baergen and Heller, 2020; Buonsenso D et al., 2020; Yu N, Li, Kang, Zeng, et al., 2020; Juusela et al., 2020; Silverstein et al., 2020; Yassa et al., 2020a; Lucarelli et al., 2020; Govind et al., 2020; Cooke et al., 2020; Dória et al., 2020; Xu et al., 2020; Cao et al., 2020; Andrikopoulou et al., 2020; Huang W et al., 2020; Breslin et al., 2020). The 31 case series identified 211 pregnant women (mean ± SD; age 31.4 ± 5.5 years; gestational age 35.7 ± 3.8 weeks). Concerning women’s medical history, diabetes (n=18), hypertension (n=15), asthma (n=6), anemia (n=4), hypoxia (n=3), hypothyroidism (n=2), influenza (n=1), polycystic ovary syndrome (n=1), gestational cholecystitis (n=1), placenta previa (n=1), septic shock (n=1), hepatitis B (n=1), anorexia (n=1), vaginal bleeding (n=1), psoriasis (n=1), scoliosis (n=1), severe myopia (n=1) and liver dysfunction (n=1) were recorded (Table 2 in Supplementary Data^1^). All 211 women were confirmed COVID-19 cases through RT-PCR or presented positive findings in CT.

#### Data about delivery in case series:

From 211 pregnant women, 83% (175/211) gave birth, while the remaining were still pregnant during the study period. Concerning the mode of delivery, 130 women (74.2%, 130/175) were subjected to C-section and 45 women (25.8%, 45/175) delivered through vaginal route. The most common indication for C-section was COVID-19 pneumonia (25.4%, 33/130), followed by fetal distress (10%, 13/130) and pre-eclampsia (6.9%, 9/130), while indications were not reported in 43.1% of cases (Table 2 in Supplementary Data^1^).

#### Therapeutic management in case series:

During hospitalization, information about treatment was provided for 70 out of 211 cases (33.2%); in the other 66.8% of cases, treatment-related information was not reported. Hydroxychloroquine (28.5%, 20/70), azithromycin (25.7%, 18/70), lopinavir/ritonavir (21.4%, 15/70), oseltamivir (14.2%, 10/70), ceftriaxone (12.8%, 9/70), and methylprednisolone (7.1%, 5/70) were administered more often. Most women were treated with antivirals and antibiotics and 29 of them (41.4%, 29/70) needed O2 support; 24 women were transferred to ICU (11.4%, 24/211) due to respiratory deterioration and COVID-19 complications (Table 2 in Supplementary Data^1^).

#### Neonatal and maternal outcomes in case series:

A total of 179 neonates were born, including 4 pairs of twins (4.5%, 8/179); 173 were born alive and 6 died (3.3% 6/179). Causes of deaths included refractory shock, multiple organ failure, intrauterine fetal death (n=2) and pre-eclampsia (n=2) of the mother. In many cases neonates were tested for SARS-CoV-2 shortly after birth; 101 were negative (92.7%, 101/109) and 8 were positive (7.3%, 8/109). 70 neonates were not tested or no test was reported (43.6%, 70/179). Most of the neonates were healthy, with some exceptions including fetal growth restriction (n=7), premature rupture of membranes (PROM) (n=3), respiratory distress syndrome (RDS) (n=2), mild pneumonia (n=2), fever (n=1), tachypnea (n=1), asphyxia (n=1), fetal growth discordance (n=1), spontaneous bowel perforation (n=1), talipes (n=1), pyrexia (n=1), cyanosis (n=1) and admission to NICU (n=11) due to precautionary measures for COVID-19 and prematurity (Table 2 in Supplementary Data^1^).

Nine pregnant women presented pneumonia, with respiratory symptoms in most cases (4.3%, 9/211). Twenty-six women were in good health (26.5%, 56/211), twenty-eight were discharged from hospital (13.3%, 28/211), while no data were reported regarding the health of 105 women (49.7%, 105/211). Some women needed more care, including ventilator support (n=1), support by extracorporeal membrane oxygenation (ECMO) (n=1), ICU (n=1) and re-admission (n=3) in hospital. Seven deaths were reported in mothers (3.3%, 7/211) due to COVID-19 complications (Table 2 in Supplementary Data^1^).

### Cohort Studies

#### Demographics in cohort studies:

The 40 cohort studies identified 5,242 pregnant women, from which 2,685 (51% 2,685/5,242) were confirmed cases of COVID-19 through RT-PCR or CT scan (Table 3 in Supplementary Data^1^) and were included in the present study (Chen et al., 2020; Liu et al., 2020; Liu et al., 2020; Liu et al., 2020; Yan et al., 2020; Wu et al., 2020; Shanes et al., 2020; Ferrazzi et al., 2020; Penfield et al., 2020; Collin et al., 2020; Qadri and Mariona, 2020; Qiancheng et al., 2020; Wu et al., 2020; Pierce-Williams et al., 2020; Yang et al., 2020; Kayem et al., 2020; Knight et al., 2020; Khalil et al., 2020; Chen et al., 2020; Lokken et al., 2020; Liao et al., 2020; London et al., 2020; Patanè et al., 2020; Savasi et al., 2020; Pereira et al., 2020; Khoury et al., 2020; Zeng Y et al., 2020; Sentilhes et al., 2020; Zeng Q-L et al., 2020; Mendoza et al., 2020; Wang et al., 2020; Martínez-Perez et al., 2020; Miller et al., 2020; Blitz et al., 2020; Fox and Melka, 2020; Campbell et al., 2020; Emeruwa et al., 2020; Gagliardi et al., 2020; Goldfarb et al., 2020). The age range of women was 18-41 years and the gestational age ranged from 5 to 40 weeks. The majority of studies derived mainly from China (35%, 14/40) and US (35%, 14/40), 4 from Italy (10%, 4/40), 3 from Spain (7.5%, 3/40), 2 from UK (5%, 2/40), 2 from France (5%, 2/40) and 1 from Sweden (2.5%, 1/40), as shown in Table 3 in Supplementary Data^1^. Co-morbidities were reported in 249 pregnant women and included gestational diabetes (22.8%, 57/249), obesity (21.6%, 54/249), hypertension (20.8%, 52/249), hypothyroidism (14.4 %, 36/249), asthma (8.0%, 20/249), and hepatitis B (2%, 5/249).

#### Data about delivery in cohort studies:

In cohort studies, 1,789 neonates were born, including 3 pairs of twins (0.3%, 6/1789) from 1,786 pregnancies. In 378 cases the delivery mode was not mentioned; in the remaining 1,408 pregnancies 929 C-sections (66.0%, 929/1,408) and 479 vaginal deliveries were reported (34.0%, 479/1408). Indicators for C-section were mainly related to COVID-19 symptoms (8.1%, 75/929), fetal distress (3.6%, 34/929) and fetal heart complications (2.4%, 23/929) (Table 3 in Supplementary Data^1^).

#### Therapeutic management in cohort studies:

Treatment details were recorded in 1,267 cases; 25.8% (327/1,267) of pregnant women received antiviral therapy, 18.2% (231/1,267) O2 support, 19.7% (250/1,267) antibiotics, 16.2 % (206/1,267) corticosteroids, 6.4% (82/1,267) hydroxychloroquine, 2.2% (29/1,267) remdesivir, 1.6% (21/1,267) azithromycin, 1%, (13/1,267) interleukin-6 inhibitors, 1%, (13/1,267) convalescent plasma, 0.6% (8/1,267) oseltamivir, 0.3% (4/1,267) lopinavir/ritonavir, and 46 were admitted to ICU (3.6%, 46/1,267) (Table 3 in Supplementary Data^1^).

#### Neonatal and maternal outcomes in cohort studies:

The health status was reported in 316 neonates from a total of 1,789 cases, with 62.2% being admitted to neonatal intensive care unit (NICU) (197/316), 18.6% being completely healthy (59/316), 15.8% being discharged from hospital shortly after birth (50/316), 0.3% presenting with pneumonia (1/316) and 0.3% remaining in hospital (1/316). Neonatal deaths were reported in 2.5% of cases (8/316); five due to COVID-19 (5/316, 1.6%), one to neonatal asphyxia, one to prematurity and one to intrauterine fetal death (IUFD). The majority of neonates (97.1%, 698/719) were tested negative for SARS-CoV-2; 21 were tested positive (2.9%, 21/719); the remaining 1,070 were either not tested or relevant data were not reported (59.8%, 1,070/1,789) (Table 3 in Supplementary Data^1^).

Maternal health outcome was reported in 779 cases. Forty-four pregnant women remained hospitalized (5.6%, 44/779) during the study period, 80.6% were discharged (629/779) and 12.3% were in good health (96/779). One woman needed ventilation and one plasmapheresis. Eight women died (1%, 8/779), due to COVID-19 complications, multiple organ failure and severe respiratory distress (Table 3 in Supplementary Data^1^).

### Case-control studies

Two case-control studies were identified (Li N et al., 2020; Tekbali et al., 2020). One study in New York City, in March 2020, compared 3,064 pregnant with 18,916 non-pregnant control women concerning COVID-19-related admission to hospitals. The rates of admission of pregnant/postpartum and control women increased from week 1 to week 4 of the COVID-19 outbreak from 0.14% to 5.65% and from 1.21% to 56.79%, respectively (Table 4 in Supplementary Data^1^).

Another study, conducted in Wuhan, in January to February 2020, compared 34 pregnant women with COVID-19 (n=16) or suspected COVID-19 (n=18) with 121 pregnant women without COVID-19 and 121 pregnant women who had been admitted for other reasons in the past (2019). The COVID-19 group gave birth to 30 neonates (all via C-section) and the control group to 101 neonates (all via C-section). Concerning the health of the pregnant women, three presented with gestational diabetes (8.8%, 3/34), three with gestational hypertension (8.8%, 3/34), one with hypothyroidism (2.9%, 1/34), one with sinus tachycardia (2.9%, 1/34) and one with pre-eclampsia (2.9%, 1/34), and were all discharged home. The main therapeutic treatment administered was antibiotics (100%, 34/34) and antivirals (11.7%, 4/34). No complications in neonatal health were mentioned and all neonates were negative for SARS-CoV-2 (n=30) (Table 4 in Supplementary Data^1^).

#### Risk of bias:

According to Newcastle-Ottawa scale ratings the majority of the 40 cohort studies were identified as good or fair quality studies (11/40 and 17/40, respectively). Eleven studies were scored as poor quality (Breslin et al., 2020; McLaren et al., 2020; Liu H et al., 2020; Liu W et al., 2020; Ferrazzi et al., 2020; Khalil et al., 2020; Chen L et al., 2020; Lokken et al., 2020; Martínez-Perez et al., 2020; Miller et al., 2020) . Short follow-up period was the most prominent factor compromising the quality of studies. Most studies presented clear inclusion criteria, with detailed description of the sample (pregnant women exposed to SARS-CoV-2, randomly selected) while data were derived from reliable, hospital records. Unadjusted (univariate) estimates were provided as a rule; almost half of the studies included a non-exposed group that matched with the exposed group in factors, such as age, gestational age, delivery mode and comorbidities. In the studies of poor quality, there was no description of the non-exposed group or any comparison with the exposed one.

## Discussion

COVID-19 is a global health issue, several months after the onset of the outbreak. Pregnant women are considered a high risk group, not only physically, but psychologically as well (Birkeland and Kristoffersen, 1980; Goodnight and Soper, 2005; Kotabagi et al., 2020; Ifdil et al., 2020; Zeng L-N et al., 2020; Masjoudi et al., 2020; Wu, Zhang et al., 2020; Lee T-Y et al., 2020; Suzuki, 2020; Huang J-W et al., 2020; Berthelot et al., 2020; Durankuş and Aksu, 2020; Yassa et al., 2020b; Corbett et al., 2020). As new data concerning the virus and its transmission are constantly emerging, the present systematic review comprises various types of studies and a considerably larger sample (8,092 patients, 6,046 pregnant women and 2,046 neonates) compared to previous efforts.

There has been a great controversy conserning the vertical transmission of SARS-CoV-2 between positive mothers and embryos. In the present systematic review, the majority of neonates were tested negative (case reports 92.7%, case series studies 84.2%, cohort studies 97.1% and case control studies 100%), while all mothers were tested positive. Our findings are in line with the literature (Dong L et al., 2020; Carosso et al., 2020; Kirtsman et al., 2020; Zeng L et al., 2020; Ferrazzi et al., 2020; Kayem et al., 2020) supporting that vertical transmission does not occur in the majority of neonates (Wang S et al., 2020; Dong L et al., 2020; Wen et al., 2020; Wang X et al., 2020; Bani Hani et al., 2020; Rabice et al., 2020), as there are only a few reports of potential transplacental transmission of the virus (Vivanti et al., 2020). Most studies have shown that there were no clinical findings of COVID-19 in neonates born to affected mothers and all samples concerning amniotic fluid, cord blood, placentas and breast milk, were negative (Chen S., Liao, et al., 2020; Liu Y et al., 2020; Zhu et al., 2020; Breslin et al., 2020; Chen, Zhang et al., 2020). In cases where the neonates were tested positive, the virus might have been transmitted in other ways, such as with touch, droplets or breast milk (Buonsenso et al., 2020); therefore, the value of universal screening of women admitted for delivery has been supported, especially because many positive women are asymptomatic. Thus, it is of paramount importance to screen pregnant women before labor (Sutton et al., 2020; Fassett et al., 2020).

Caesarean section has been the most common mode of delivery since the start of the COVID-19 epidemic and especially in China, with a rate over 90% (Yang H et al., 2020). A previous systematic review reported that about 75% of the infected women delivered by C-section (Corbett et al., 2020). According to the present systematic review, a substantial proportion of pregnant women with COVID-19 underwent C-section (case reports 82.2%, case series 74.2% and cohort studies 66.0%). Recent guidelines suggest C-section to be considered in cases of severe and critical infections while taking into account possible risks (Royal College of Obstetricians and Gynecologists, 2020). On the other hand, a study from Spain reported that deliveries by C-section were significantly associated with clinical deterioration of positive mothers (Martínez-Perez et al., 2020). Additionally, there is no evidence that the rate of neonatal COVID-19 is lower when the baby is born by C-section (Walker et al., 2020), hence, C-section could be applied in cases where other indications also exist (Ashokka et al., 2020).

In many studies the reason for C-section was not mentioned. It is possible that, because COVID-19 complications are not well-known, especially in the vulnerable group of pregnant women and neonates, increased anxiety of both mothers and doctors might have led to rash decisions. Actually, as already mentioned, C-section was the rule in China and other countries during the first months of the pandemic (Martínez-Perez et al., 2020).

According to Zaigham and Andersson (Zaigham and Andersson, 2020), COVID-19 is a risk factor for increased maternal and perinatal morbidity, probably due to higher rates of preterm birth in mothers with COVID-19 (Allotey et al., 2020). Two maternal deaths and only one neonatal death were reported in a recent systematic review, including 324 pregnant women (Juan et al., 2020). Our study comprised a larger sample with mortality rates ranging from 1% in cohort studies to 5.7% in case reports in mothers, and from 2% in case reports to 3.3 % in case series in neonates. Further studies are needed to estimate standardized mortality ratios in COVID-19 pregnant women and their neonates versus pregnant control women, addressing the confounding effects of comorbidities, as pre-existing comorbidities of the mother such as advanced maternal age and high body mass index are potential risk factors for severe COVID-19 during pregnancy (Allotey et al., 2020).

The main limitation of the present systematic review is that most of the currently available studies did not provide detailed data for participants, probably due to the emergency nature of the subject. Additionally, a substantial amount of evidence was derived from case reports and case series. Moreover, the inadequacy of follow-up periods reduced the validity of cohort studies. Finally, the lack of important data in many studies, such as the positivity of neonates in SARS-CoV-2 testing, mode of delivery and indication for C-section, did not allow extensive analyses.

On the other hand, this systematic review has several strengths as it includes a large sample with detailed data about the mode of labour, morbidity, and vertical transmission. Case reports and case series highlighted important aspects of the disease. Moreover, studies from all continents, except Africa, were included, whereas existing systematic reviews refer mostly to studies derived from China.

In conclusion, according to the present systematic review, vertical transmission of COVID-19 from mother to child is rare. Nevertheless, careful screening of pregnant women seems important in view of adverse health outcomes for the mother and the neonate. Specific guidelines with evidence-based decision algorithms for the mode of delivery in the context of a pregnancy affected by COVID-19 are needed.

## Supplementary Material

Tables 1-4 in Supplementary Data

## Key Points

- Vertical transmission of SARS-CoV-2 from mother to child seems to be rare.
- A substantial proportion of pregnant women with COVID-19 underwent caesarean section.
- Maternal mortality rates ranged from 1% to 5.7% and neonatal mortality from 2% to 3.3%.
- Specific guidelines in the context of a pregnancy affected by COVID-19 are needed.

## Footnotes

1
http://journal.embnet.org/index.php/embnetjournal/article/downloadSuppFile/969/969_supp_1
