# Supplementary material for: The impact of COVID-19 during pregnancy on maternal and neonatal outcomes: a systematic review
Source: EMBnet J. Author manuscript; Available in PMC 2022 May 19. (PMC9119021; doi:10.14806/ej.26.1.969)
Supplement: Tables 1-4 in Supplementary Data [file NIHMS1766877-supplement-Tables_1-4_in_Supplementary_Data.docx]

Table 1: Case reports of pregnancies affected by COVID-19

| **First author (year)** | **Region, country** | **Study period** | **Gestational age** | **Maternal age**  **(years)** | **Test for**  **Covid-19** | **Mode of delivery** | **Indication for**  **C-section** | **Treatment received** | **Newborn health** | **SARS-CoV-2 status of the newborn** | **Maternal comorbidities** | **Maternal health at the end of study** | **Special remarks** |
| --- | --- | --- | --- | --- | --- | --- | --- | --- | --- | --- | --- | --- | --- |
| Wang et al., (2020) | Wuhan, China | Feb 1-Feb 18 | 40w | 34 | CT scan (positive) | C-section | NR | antiviral, antibiotic, methylprednisolone | good | positive (36h) | hypothyroidism | good | none |
| Dong et al.,  (2020) | Wuhan China | Jan 28-Feb 28 | 34w+2 | 29 | RT-PCR  CT scan (positive) | C-section | NR | O_2_, antiviral, antibiotic, corticosteroid | NR | IgM 2 hours after birth | none | NR | possible neonatal infection in uterus, however nasopharyngeal swabs negative |
| Wang et al.,  (2020) | Suzhou China | Feb 2 | 30w | 28 | RT-PCR (positive) | C-section | fetal distress | antiviral, human albumin, dexamethasone | good | negative | NR | NR | none |
| Li et al.,  (2020) | Hangzhou, China | Feb 6-Feb 24 | 35w | 30 | CT scan (positive) | C-section | fetal distress | antiviral, methylprednisolone | good | negative | NR | good | none |
| Xiong et al., (2020) | Beijing China | Jan 29- March 10 | 33w+1 | 25 | RT-PCR (positive) | vaginal delivery | - | NR | good | negative | NR | good | none |
| Liao et al., (2020) | Chongqing China | Feb 9 | 35w+1 | 25 | RT-PCR (positive) | C-section | fetal distress | antibiotic, antiviral | NR | negative | NR | NR | none |
| Chen Rong et al.,  (2020) | China | NR | 36w | 27 | RT-PCR (positive) | C-section | concern about progression of COVID-19 pulmonary manifestations | NR | NR | negative | NR | NR | none |
| Peng et al., (2020) | China | NR | 35w+3 | 25 | RT-PCR  CT scan (positive) | C-section | fetal distress | interferon, lopinavir, antibiotics, O_2_, dexamethasone for fetal lung development | ICU | negative | NR | NR | none |
| Li J et al., (2020) | China | Feb 1-March 17 | 35w+2 | 31 | NR (positive) | C-section | emergency situation | antivirals, antibiotics, O_2_, glucocorticoids, ventilation, immunotherapy, blood transfusion, traditional Chinese medicine | died 2 hours after delivery | NR | none | discharged | possible COVID-19 related neonatal death due to inflammatory storm, which may also attack fetal organs |
| Lang et al., (2020) | China | Feb 1-Feb 19 | 35w+2 | 30 | RT-PCR (positive) | C-section | fetal distress | NR | good | negative | NR | discharged | none |
| Yu & Fan (2020) | Wuhan, China | January 28, 2020 | 34w | 35 | RT-PCR  CT scan (positive) | vaginal delivery | - | cephalosporin, imipenem, moxifloxacin, linezolid, moxifloxacin, meropenem, polymyxin b, cephalosporin, ganciclovir, albidol, interferon, micafinen voriconazole, methylprednisolone, prednisolone, thymalfasin, ventilation | ICU | negative | none | NR | none |
| Dong et al., (2020) | China | February 26, 2020 | 38w+2 | 33 | RT-PCR (positive) | NR | NR | azithromycin, ornidazole | ICU | negative | none | transferred to designated hospital for COVID-19 treatment in Wuhan city | breastfeeding may protect infants against infection |
| Du & Wang (2020) | Shaanxi, China | NR | 36w+2 | 30 | RT-PCR (positive) | emergency C-section | fetal heart rate deceleration | NR | good | NR | NR | discharged at day 5 | none |
| Xia et al., (2020) | China | Jan 20,2020 | 36w+5 | 27 | RT-PCR (positive) | C-section | oligohydramnios, intrauterine distress | O_2_, Cefazolin | NR | NR | NR | NR | none |
| Iqbal et al., (2020) | Washington | NR | 39w | 34 | RT-PCR  CT scan (positive) | vaginal delivery | - | NR | good | NR | NR | good | none |
| Zambrano et al.,  (2020) | Honduras Central America | March 9 - March 20, | 31w | 41 | RT-PCR (positive) | vaginal delivery | - | NR | hospitalized | negative | gestational hypertension, hypothyroidism | NR | none |
| Schnettler et al., (2020) | Ohio | March 24 | 31w | 39 | RT-PCR  CT scan (positive) | elective C-section | previous C-section | O_2_, corticosteroids, ceftriaxone, azithromycin, oseltamivir, hydroxychloroquine | ICU | negative | mild myotonic dystrophy | hospitalised at the end of the study | none |
| Anderson et al (2020) | Nashville, USA | NR | 22w+2 | 35 | NR (positive) | NR | NR | COVID-19 convalescent plasma transfusion, ceftriaxone, azithromycin, hydroxychloroquine, antenatal steroids, remdesivir | ICU | NR | type 2 DM, asthma, class III obesity | discharged with no further issues | none |
| Mehta et al., (2020) | Livingstone, USA | NR | 27w | 39 | RT-PCR  CT scan (positive) | C-section | maternal decompensation | ceftriaxone, azithromycin, hydroxychloroquine, betamethasone | ICU | A. female positive B. male negative | NR | discharged home | none |
| Rabice et al., (2020) | Denver, USA | NR | 33w | 36 | RT-PCR  CT scan (positive) | elective C- section | pre-eclampsia | acetaminophen, dicloxacillin, antiemetics, pantoprazole, fentanyl for pain control | good | NR | type 1 DM, mild intermittent asthma, maternal obesity (BMI 44 kg/m^2^),  pre-eclampsia | NR | none |
| Sinkey RG et al., (2020) | Alabama | March 2020 | 39w | 25 | RNA-PCR (positive) | C-section | NR | antihypertensives | NR | NR | hypertension, pulmonary edema, elevated brain natriuretic peptide, and high-sensitivity troponin-I | SARS-CoV-2 positive 6 days after discharge | none |
| Hong et al., (2020) | Detroit, MI, USA | NR | 23w | 36 | NR (positive) | C-section | pneumonia | hydroxychloroquine, prednisone, betamethasone for fetal lung immaturity | ICU | NR | hypothyroidism, morbid obesity, hyperlipidemia | successfully discharged stable on day 11 | none |
| Blauvelt et al., (2020) | San Francisco, California | NR | 28w+4 | 34 | RT-PCR (positive) | C-section | pneumonia | hydroxychloroquine, azithromycin, ceftriaxone, betamethasone (fetal lung immaturity), remdesivir, oxytocin and methergine (uterine atony), prednisone, ertapenem, ventilation | ICU | negative | moderate asthma, gestational DM, class II obesity, and three prior  C-sections | discharged home in good condition on postoperative day 15 | none |
| Kelly et al., (2020) | St. Louis, MO | NR | 33w | NR | RT-PCR  CT scan (positive) | C-section | persistent maternal tachycardia and high fever, and fetal heart rate deceleration | antibiotics | ICU | negative | NR | hospitalized | none |
| Ahmed et al., (2020) | UK | January-April 8, 2020 | 31w | 29 | RT-PCR (positive) | C- section | NR | corticosteroids (fetal lung immaturity), amoxicillin, enoxaparin (venous thromboembolism prophylaxis) | ICU | negative | type 2 DM treated with metformin and insulin, renal tubular acidosis, asthma, vitamin D deficiency | death due to COVID-19 and artery thrombosis | both pregnancy and COVID-19 increase the risk of thrombosis; this case highlighted the synergy of these factors in increasing the risk of thrombotic complications in pregnant women with COVID-19, especially those admitted to ICU |
| Nesr et al., (2020) | UK | NR | 40w | 34 | PCR (positive) | NR | NR | intravenous immunoglobulins, prednisolone | NR | NR | immune thrombocytopenia (ITP) | NR | none |
| Kuhrt et al., (2020) | UK | March 13-April 2, 2020 | 32w+6 | 30 | PCR (positive) | emergency C-section | antepartum hemorrhage with placental abruption | antenatal corticosteroids for fetal lung immaturity | twins - ICU | negative/negative | thyroidectomy | Postnatally, maternal thrombocytopenia and lymphocytopenia associated with COVID-19, abnormal chest x-rays, mild pericardial effusion | none |
| AlZaghal et al., (2020) | Irbid, Jordan | March 2020 | 36w | 30 | PCR (positive) | C-section | placental abruption | hydroxychloroquine | good | negative | previous stillborn infant | discharged | none |
| Bani et al., (2020) | Irbid, Jordan | March 28, 2020 | 37w+4 | 29 | PCR (positive) | elective C- section | previous C-section | NR | good | negative | - | NR | none |
| Lee et al.,  (2020) | Korea | March 6-March 11 | 37w+6 | 28 | RT-PCR  CT scan (positive) | C-section | cephalopelvic distortion | NR | good | negative | none | good | significant hypotension following spinal anesthesia |
| Oh J et al., 2020 | Daegu, Korea | Feb 26-Aprl 3,2020 | 37w+6 | 28 | RT-PCR (positive) | C-section | cephalopelvic distortion | NR | good | negative | - | NR | NR |
| De Socio et al., (2020) | Perugia, Italy | March 30, 2020 | 40w | 33 | RT-PCR (positive) | vaginal delivery | - | oxytocin | good | negative | none | discharged 2 days after delivery | none |
| Carosso et al., (2020) | Turin, Italy | NR | 37w | 28 | PCR (positive) | vaginal delivery | - | NR | ICU | positive | gestational DM | NR | none |
| Martinelli I et al (2020) | Milan, Italy | March 29, 2020 | 29w | 17 | RT-PCR  CT scan (positive) | emergency C-section | dyspnea | enoxaparin, enoxaparin (antithrombotic prophylaxis) | ICU | NR | obesity | NR | COVID-19 and Staphylococcus aureus infection developed pulmonary embolism |
| Mohammadi et al., (2020) | Sanandaj, Iran | May 2, 2020 | 8w | 26 | RT-PCR (positive) | NR | NR | NR | NR | NR | complex obesity and respiratory failure | NR | acute thrombosis during COVID-19 disease |
| Zamaniyan et al., (2020) | Sari, Iran | March 7-March 25 | 32w | 22 | q-PCR,  CT scan (positive) | C- section | NR | azithromycin, ceftriaxone, lopinavir/ritonavir, oseltamivir, hydroxychloroquine | ICU | first test negative, second test after 24h positive, third and fourth tests after 1 week positive | hypothyroidism | maternal death at day 19th due to COVID-19 | amniotic fluid sample positive for SARS-CoV-2 |
| Taghizadeh et al., (2020) | Iran | NR | 34w | 33 | PCR (positive) | C-section | maternal renal condition | lopinavir/ritonavir, ribavirin, oseltamivir, meropenem, ciprofloxacin, vancomycin | good | negative | none | ICU | none |
| Kalafat  (2020) | Ankara Turkey | March 20-March 28 | 35w+3 | 32 | RT-PCR  CT scan (positive) | C-section | NR | O_2_, azithromycin, hydroxychloroquine, antiviral | ICU | negative | thalassemia trait | ICU | none |
| Yilmaz et al., (2020) | Konya, Turkey | NR | 38w | 25 | RT-PCR (positive) | C-section | NR | hydroxychloroquine, oseltamivir, azithromycin | good | negative | NR | good | none |
| Alzamora et al.,  (2020) | Lima Peru | March 19 | 33w | 41 | RT-PCR (positive) | C-section | maternal respiratory status | azithromycin, hydroxychloroquine, meropenem, vancomycin, oseltamivir | NR | positive at 16 hours | DM | NR | none |
| Wen et al.,  (2020) | Qingdao Shandong | Feb 4-Feb 20 | 30w | 31 | RT-PCR (positive) | no delivery | - | interferon, antiviral | none | NR | NR | cured | none |
| Lowe et al., (2020) | Australia Queensland | NR | 40w+3 | 31 | RT-PCR (positive) | vaginal delivery | - | gentamycin, metronidazole, cephazolin (possible chorioamnionitis) | good | negative | NR | NR | none |
| Lu et al., (2020) | Ireland | NR | 40w | 29 | RT-PCR (positive) | C- section | maternal fever, fetal tachycardia, maternal sepsis and previous C- section | NR | good | NR | chronic hepatitis B infection | afebrile on day 2 of hospitalization and discharged home with thromboprophylaxis | none |
| Lyra et al., (2020) | Portugal | NR | 39w+6 | 35 | RT-PCR (positive) | C-section | spontaneous PROM | NR | good | negative | NR | discharged home at 48 hours postpartum | none |
| Diaz et al., (2020) | Spain | NR | 38w+4 | 41 | RT-PCR (positive) | emergency C- section | severe pre-eclampsia | ICU | ICU | negative, positive after 9 days | hypothyroidism | NR | none |
| Fontanella et al., (2020) | Netherlands | NR | 31w+6 | 38 | RT-PCR (positive) | NR | NR | antenatal corticosteroids for fetal lung maturation, hydroxychloroquine | NR | NR | gestational DM | discharged after 5 days of rapid improvement | none |
| Piersigilli et al., (2020) | Belgium | March 1, 2020 | 40w | 29 | RT-PCR (positive) | C-section | maternal fever, fetal tachycardia, maternal sepsis, previous C- section | NR | good | negative | hepatitis B | discharged | none |
|  |  |  | 26w+4 | NR |  | C-section | maternal HELLP | intravenous antibiotics, corticosteroids for fetal lung immaturity | still in ICU |  | pre-eclampsia and suspected cholecystitis | negative on day 21 after delivery |  |
| Baud et al., (2020) | Switzerland | March 2020 | 19w | 28 | RT-PCR (positive) | miscarriage | - | acetaminophen, prophylactic amoxicillin-clavulanic acid | NR | negative | obesity | NR | none |
| Gidlof et al.,  (2020) | Stockholm Sweden | March | 36w+2 | 34 | RT-PCR (positive) | C-section | hypertension | paracetamol, labetalol, nifedipine, antibiotic | twin 1 good  twin 2 good | both negative | hypertension, proteinuria, gestational DM | NR | none |
| Kirtsman et al., (2020) | Toronto, Ontario | NR | 35w+5 | 40 | RT-PCR (positive) | semi-emergency C-section | coagulopathy (elevated d-dimers, fibrinogen, and activated partial thromboplastin  time) | NR | ICU | positive | familial neutropenia, gestational DM, history of frequent bacterial infections during pregnancy | discharged on day 4 | none |
| Panichaya et al., (2020) | Thailand | NR | 18w | 43 | RT-PCR (positive) | pregnancy termination | - | no treatment | termination of pregnancy | negative | amniocentesis due to high risk of aneuploidy | clinically improved without fever | fetal Down syndrome |

*COVID-19: coronavirus disease 2019, w: weeks, C-section: caesarian cection, NR: not reported, ICU: intensive care unit, CT: computed tomography, q-PCR: quantitative polymerase chain reaction, RT-PCR: reverse transcription polymerase chain reaction, O_2_: oxygen,* *PROM*: *premature rupture of membranes, DM: diabetes mellitus, BMI: body mass index*

Table 2. Case series of pregnancies affected by COVID-19

| **First author (year)** | **Region, country** | **Study period** | **Sample size** | | **Test for COVID-19** | **Gestational age** | **Maternal age (years)** | **Mode of delivery** | **Indication for**  **C-section** | **Treatment received** | **Newborn health** | **SARS-CoV-2 status of the newborn** | **Maternal comorbidities** | **Maternal health at the end of study** |
| --- | --- | --- | --- | --- | --- | --- | --- | --- | --- | --- | --- | --- | --- | --- |
| Chen H et al., | Wuhan China | Jan 20-Jan 31, 2020 | 9 | patient 1 | qRT-PCR CT scan (all positive) | 37w+2 | 33 | C-section | severally elevated AST/ALT, COVID-19 pneumonia | O_2_, antiviral, antibiotic | good | not received sample | influenza | pneumonia diverse |
|  |  |  |  | patient 2 |  | 38w+2 | 27 | C-section | COVID-19 pneumonia | O_2_, antiviral, antibiotic | good | negative | none | pneumonia diverse |
|  |  |  |  | patient 3 |  | 36w | 40 | C-section | previous C-sections  COVID-19 pneumonia | O_2_, antiviral, antibiotic | prematurity | not received sample | gestational hypertension | pneumonia diverse |
|  |  |  |  | patient 4 |  | 36w | 26 | C-section | pre-eclampsia  COVID-19 pneumonia | O_2_, antibiotic | prematurity  low birth weight | negative | pre-eclampsia | pneumonia diverse |
|  |  |  |  | patient 5 |  | 38w+1 | 26 | C-section | fetal distress, COVID-19 pneumonia | O_2_, antibiotic | - | negative | none | pneumonia |
|  |  |  |  | patient 6 |  | 36w+3 | 26 | C-section | history of stillbirths  COVID-19 pneumonia | O_2_, antibiotic | prematurity | negative | none | pneumonia |
|  |  |  |  | patient 7 |  | 36w+2 | 29 | C-section | PROM  COVID-19 pneumonia | O_2_, antiviral, antibiotic | good | not tested | none | pneumonia |
|  |  |  |  | patient 8 |  | 38w | 28 | C-section | fetal distress  COVID-19 pneumonia | O_2_, antiviral antibiotic | good | negative | none | pneumonia |
|  |  |  |  | patient 9 |  | 39w+4 | 34 | C-section | PROM  COVID-19 pneumonia | O_2_, antiviral antibiotic | good | negative | none | pneumonia |
| Yu N et al., (2020) | Wuhan China | Jan 1-Feb 8, 2020 | 7 | patient 1 | RT-PCR CT scan (all positive) | 39w+6 | 34 | C-section | - | O_2_, antiviral, antibiotic, methylprednisolone | good | positive (36h) | hypothyroidism | good |
|  |  |  |  | patient 2 |  | 38w+5 | 30 | C-section | multidisciplinary team decision | O_2_, antiviral, antibiotic, methylprednisolone | good | not tested | PCOS | good |
|  |  |  |  | patient 3 |  | 41w+2 | 31 | C-section | multidisciplinary team decision | O_2_, antiviral, antibiotic, methylprednisolone | good | negative | none | good |
|  |  |  |  | patient 4 |  | 37w | 33 | C-section | increased fetal movement | O_2_, antiviral, antibiotic, methylprednisolone | good | non tested | none | good |
|  |  |  |  | patient 5 |  | 40w+4 | 29 | C-section | multidisciplinary team decision | O_2_, antiviral, antibiotic, methylprednisolone | good | non tested | none | good |
|  |  |  |  | patient 6 |  | 38w+2 | 34 | C-section | multidisciplinary team decision | O_2_, antiviral, antibiotic, | good | non tested | none | good |
|  |  |  |  | patient 7 |  | 38w+4 | 34 | C-section | multidisciplinary team decision | O_2_, antiviral, antibiotic | good | negative | none | good |
| Fan et al., (2020) | Wuhan China | Jan 26-Feb 19, 2020 | 2 | patient 1 | qRT-PCR CT scan (all positive) | 37w | 34 | C-section | NR | antiviral, antibiotic methylprednisolone, Chinese medicine | low fever, discharged | negative | none | good |
|  |  |  |  | patient 2 |  | 36w | 29 | C-section | persistent high fever | antiviral, antibiotic, Chinese medicine, methylprednisolone | mild pneumonia | negative | none | good |
| Liu et al., (2020) | outside Wuhan | Dec 8 2019- Feb 25 2020 | 13 | patient 1 | RT-PCR (all positive) | 25w | 28 | No delivery | NR | NR | NR | NR | none | good |
|  |  |  |  | patient 2 |  | 27w | 24 | No delivery | NR | NR | NR | NR | none | good |
|  |  |  |  | patient 3 |  | 32w | 33 | C-section | NR | NR | good | negative | none | good |
|  |  |  |  | patient 4 |  | 33w | 29 | No delivery | NR | NR | NR | NR | none | good |
|  |  |  |  | patient 5 |  | 34w | 35 | C-section | PROM | NR | good | negative | none | good |
|  |  |  |  | patient 6 |  | 34w | 31 | C-section | NR | ICU | MODS stillbirth | negative | none | still in ECMO |
|  |  |  |  | patient 7 |  | 35w | 30 | C-section | fetal distress | NR | good | negative | none | good |
|  |  |  |  | patient 8 |  | 35w+5 | 36 | C-section | NR | NR | good | negative | none | good |
|  |  |  |  | patient 9 |  | 36w | 26 | C-section | NR | NR | good | negative | none | good |
|  |  |  |  | patient 10 |  | 36w+4 | 32 | C-section | fetal distress | NR | good | negative | none | good |
|  |  |  |  | patient 11 |  | 37w | 30 | C-section | fetal distress | NR | good | negative | none | good |
|  |  |  |  | patient 12 |  | 38w | 22 | C-section | NR | NR | good | negative | none | good |
|  |  |  |  | patient 13 |  | 38w+3 | 30 | C-section | NR | NR | good | negative | none | good |
| Zeng et al., (2020) | Wuhan | NR | 3 | patient 1 | RT-PCR (all positive) | 40w | neonates | C-section | meconium stained amniotic fluid, maternal pneumonia | NR | good | positive | NR | NR |
|  |  |  |  | patient 2 |  | 40w+4 | neonates | C-section | maternal pneumonia | NR | good | positive | NR | NR |
|  |  |  |  | patient 3 |  | 31w+2 | neonates | C-section | fetal distress,  maternal pneumonia | NR | good | positive | NR | NR |
| Zhu et al., (2020) | Wuhan China | Jan 20-Feb 5 2020 | 9 | patient 1 | RT-PCR (all positive) | 38w +4 | 25 | C-section | NR | NR | good | negative | NR | NR |
|  |  |  |  | patient 2 |  | 33w+6 | 35 | C-section | NR | NR | good | negative | NR | NR |
|  |  |  |  | patient 3 |  | 34w+2 | 35 | vaginal | NR | NR | good | negative | NR | NR |
|  |  |  |  | patient 4 |  | 34w+5 | 30 | C-section | NR | NR | died on day 9 | negative | NR | NR |
|  |  |  |  | patient 5 |  | 39w | 30 | C-section | NR | NR | good | negative | NR | NR |
|  |  |  |  | patient 6 |  | 37w | 30 | C-section | NR | NR | in hospital | negative | NR | NR |
|  |  |  |  | patient 7 |  | 34w+6 | 30 | C-section | NR | NR | in hospital | negative | NR | NR |
|  |  |  |  | patient 8 |  | 31w | 29 | vaginal (twins) | NR | NR | in hospital | negative | negative result despite pneumonia on CT scan | NR |
|  |  |  |  | patient 9 |  | 39w | 34 | C-section | NR | NR | in hospital | negative | NR | NR |
| Chen et al., (2020) | Wuhan China | Jan 20-Feb 10 2020 | 5 | patient 1 | qRT-PCR (all positive) | 40w+4 | 29 | vaginal | NR | NR | good | negative | gestational DM | good |
|  |  |  |  | patient 2 |  | 39w+1 | 30 | C-section | fetal tachycardia | NR | good | negative | pre-eclampsia | good |
|  |  |  |  | patient 3 |  | 38w+6 | 25 | vaginal | NR | NR | good | negative | none | good |
|  |  |  |  | patient 4 |  | 39w+6 | 31 | vaginal | NR | NR | good | negative | none | good |
|  |  |  |  | patient 5 |  | 39w | 29 | C-section | elective C-section | NR | good | negative | gestational DM | good |
| Chen Y et al., (2020) | Wuhan | Feb 22 2020 | 4 | patient 1 | RT-PCR (all positive) | 37w+2 | 28 | C-section | NR | NR | Good | negative | NR | good |
|  |  |  |  | patient 2 |  | 39w | 34 | C-section | NR | NR | skin rash after birth – ICU | negative | cholecystitis | good |
|  |  |  |  | patient 3 |  | 37w+3 | 23 | C-section | NR | NR | ICU | negative | placenta previa | good |
|  |  |  |  | patient 4 |  | 38w+4 | 31 | vaginal | NR | NR | good | negative | NR | good |
| Yang et al., (2020) | China | Jan 20-Jan 29, 2020 | 7 | Patient 1 |  | 37w+2 | NR | C-section | 4 cases C-section to prevent transmission to newborns, 2 cases emergency C-section for hypertension and blurred vision caused by severe pre-eclampsia and 1 case C-section because of elevated aminotransferase | 2 premature infants on CPAP,  4 newborns on piperacillin, tazobactam | ICU | negative | 1 mother had hypertension  1 mother had pre-eclampsia 1 mother had elevated aminotransferase | NR |
|  |  |  |  | Patient 2 |  | 38w+2 | NR | C-section | NR |  | discharged | negative |  | NR |
|  |  |  |  | Patient 3 |  | 36w+2 | NR | C-section | NR |  | discharged | negative |  | NR |
|  |  |  |  | Patient 4 |  | 36w+3 | NR | C-section | NR |  | discharged | negative |  | NR |
|  |  |  |  | Patient 5 |  | 36w | NR | C-section | NR |  | discharged | negative |  | NR |
|  |  |  |  | Patient 6 |  | 36w+2 | NR | C-section | NR |  | discharged | negative |  | NR |
|  |  |  |  | Patient 7 |  | 38w | NR | C-section | NR |  | discharged | negative |  | NR |
| Wu C et al., (2020) | China | Jan 23-Feb 10, 2020 | 8 | patient 1 | PCR CT scan (all positive) | 39w | 28 | C-section | previous C-section | all newborns on empirical antibiotic treatment and supportive care or ventilation | NR | NR | NR | NR |
|  |  |  |  | patient 2 |  | 38w+1 | 31 | C-section | PROM |  |  |  |  |  |
|  |  |  |  | patient 3 |  | 39w+1 | 30 | C-section | pre-eclampsia |  |  |  |  |  |
|  |  |  |  | patient 4 |  | 36w+4 | 30 | C-section | PROM |  |  |  |  |  |
|  |  |  |  | patient 5 |  | 37w+6 | 30 | vaginal | NR |  |  |  |  |  |
|  |  |  |  | patient 6 |  | 40w+3 | 26 | C-section | fetal distress |  |  |  |  |  |
|  |  |  |  | patient 7 |  | 40w+4 | 29 | vaginal | NR |  |  |  |  |  |
|  |  |  |  | patient 8 |  | 33w+6 | 35 | C-section | previous C-section |  |  |  |  |  |
| Hu X et al., (2020) | China | Jan 20-Feb 20, 2020 | 7 | patient 1 | RT-PCR (all positive) | 40w | 34 | C-section | NR | only patient 4 received antiviral therapy before delivery | good | positive | 1/7 had liver disfunction | good |
|  |  |  |  | patient 2 |  | 41w+2 | 31 | C-section |  |  | good | negative |  | good |
|  |  |  |  | patient 3 |  | 38w+4 | 34 | C-section |  |  | good | negative |  | good |
|  |  |  |  | patient 4 |  | 39w+5 | 30 | vaginal |  |  | good | negative |  | good |
|  |  |  |  | patient 5 |  | 38w+2 | 33 | C-section |  |  | good | negative |  | good |
|  |  |  |  | patient 6 |  | 38w+2 | 34 | C-section |  |  | good | negative |  | good |
|  |  |  |  | patient 7 |  | 37w+2 | 33 | C-section |  |  | good | negative |  | good |
| Cao et al.,  (2020) | China | Jan 23-Feb 23, 2020 | 10 | patient 1 | qRT-PCR (all positive) | 34w+6 | 31 | elective C-section | placental abruption, pre-eclampsia | NR | prematurity | negative | NR | NR |
|  |  |  |  | patient 2 |  | 40w+3 | 29 | elective C-section | fetal distress |  | PROM | NR | NR |  |
|  |  |  |  | patient 3 |  | 38w | 30 | vaginal | NR |  | NR | NR | NR |  |
|  |  |  |  | patient 4 |  | 33w+6 | 35 | elective C-section | previous C-section |  | prematurity, PROM | negative | NR |  |
|  |  |  |  | patient 5 |  | 39w+6 | 29 | C-section | fetal distress |  | NR | negative | NR |  |
|  |  |  |  | patient 6 |  | 38w+2 | 31 | C-section | fetal distress |  | PROM | NR | NR |  |
|  |  |  |  | patient 7 |  | 34w+6 | 30 | elective C-section | twins |  | prematurity | negative | NR |  |
|  |  |  |  | patient 8 |  | 39w+1 | 29 | elective C-section | previous C-section |  | NR | NR | hypothyroidism, anemia |  |
|  |  |  |  | patient 9 |  | 39w+1 | 30 | elective C-section | pre-eclampsia |  | NR | NR | NR |  |
|  |  |  |  | patient 10 |  | 40w+5 | 29 | vaginal | NR |  | NR | NR | gestational DM |  |
| Xu L et al.,  (2020) | China | Jan 21-Feb 9, 2020 | 5 | patient 1 | RT-PCR (all positive) | 38w+6 | 34 | C-section | 39w pregnant, abdominal pain | antiviral, antibiotic | good | negative (after 8 days positive) | anorexia | NR |
|  |  |  |  | patient 2 |  | 34w+4 | 25 | C- section | viral pneumonia | antiviral, antibiotic, corticosteroid | good | negative (after 1 day positive) | NR | NR |
|  |  |  |  | patient 3 |  | 37w+3 | 23 | C-section | viral pneumonia, obstetric cause | antiviral, antibiotic | good | negative (after 8 days positive) | NR |  |
|  |  |  |  | patient 4 |  | 36w+4 | 34 | C- section | viral pneumonia | antiviral, antibiotic, corticosteroid | good | negative (after 1 day positive) | NR |  |
|  |  |  |  | patient 5 |  | 37w | 28 | vaginal | viral pneumonia | antiviral, antibiotic, corticosteroid | good | negative | vaginal bleeding |  |

| Yu N et al., (2020) | China | January 30,2020 | 2 | Patient 1 | RT-PCR CT scan (both positive) | 8w+5 | 34 | NR | NR | NR | NR | NR | NR | discharged |
| --- | --- | --- | --- | --- | --- | --- | --- | --- | --- | --- | --- | --- | --- | --- |
|  |  | February 12, 2020 |  | Patient 2 |  | 10w+1 | 27 | NR | NR | NR | NR | NR | NR | discharged |

| Baergen et al., (2020) | | Weill New York | | NR | | 20 | | patient 1 | | RT-PCR (all positive) | | 39w+6 | | 35 | vaginal | NR | NR | | NR | | NR | | NR | | NR |
| --- | --- | --- | --- | --- | --- | --- | --- | --- | --- | --- | --- | --- | --- | --- | --- | --- | --- | --- | --- | --- | --- | --- | --- | --- | --- |
|  |  |  |  |  |  |  |  | patient 2 | |  |  | 38w | | 30 | vaginal |  |  |  |  |  |  |  |  |  |  |
|  |  |  |  |  |  |  |  | patient 3 | |  |  | 40w+4 | | 29 | vaginal |  |  |  |  |  |  |  |  |  |  |
|  |  |  |  |  |  |  |  | patient 4 | |  |  | 39w+4 | | 40 | C-section |  |  |  |  |  |  |  |  |  |  |
|  |  |  |  |  |  |  |  | patient 5 | |  |  | 39w+2 | | 26 | vaginal |  |  |  |  |  |  |  |  |  |  |
|  |  |  |  |  |  |  |  | patient 6 | |  |  | 37w | | 40 | vaginal |  |  |  |  |  |  |  |  |  |  |
|  |  |  |  |  |  |  |  | patient 7 | |  |  | 38w | | 19 | vaginal |  |  |  |  |  |  |  |  |  |  |
|  |  |  |  |  |  |  |  | patient 8 | |  |  | 40w+3 | | 28 | vaginal |  |  |  |  |  |  |  |  |  |  |
|  |  |  |  |  |  |  |  | patient 9 | |  |  | 39w | | 37 | C-section |  |  |  |  |  |  |  |  |  |  |
|  |  |  |  |  |  |  |  | patient 10 | |  |  | 40w+1 | | 26 | vaginal |  |  |  |  |  |  |  |  |  |  |
|  |  |  |  |  |  |  |  | patient 11 | |  |  | 36w | | 40 | C-section |  |  |  |  |  |  |  |  |  |  |
|  |  |  |  |  |  |  |  | patient 12 | |  |  | 39w | | 38 | vaginal |  |  |  |  |  |  |  |  |  |  |
|  |  |  |  |  |  |  |  | patient 13 | |  |  | 40w | | 28 | vaginal |  |  |  |  |  |  |  |  |  |  |
|  |  |  |  |  |  |  |  | patient 14 | |  |  | 33w+2 | | 40 | C-section |  |  |  |  |  |  |  |  |  |  |
|  |  |  |  |  |  |  |  | patient 15 | |  |  | 40w | | 41 | vaginal |  |  |  |  |  |  |  |  |  |  |
|  |  |  |  |  |  |  |  | patient 16 | |  |  | 32w+2 | | 16 | vaginal |  |  |  |  |  |  |  |  |  |  |
|  |  |  |  |  |  |  |  | patient 17 | |  |  | 35w+3 | | 36 | C-section |  |  |  |  |  |  |  |  |  |  |
|  |  |  |  |  |  |  |  | patient 18 | |  |  | 39w+5 | | 23 | vaginal |  |  |  |  |  |  |  |  |  |  |
|  |  |  |  |  |  |  |  | patient 19 | |  |  | 38w+4 | | 25 | vaginal |  |  |  |  |  |  |  |  |  |  |
|  |  |  |  |  |  |  |  | patient 20 | |  |  | 37w+6 | | 32 | vaginal |  |  |  |  |  |  |  |  |  |  |
| Mclaren et al., (2020) | | Brooklyn NY | | NR | | 12 | | patient 1 | | PCR (all positive) | | 29w+4 | 44 | C-section | maternal respiratory distress | | NR | | NR | | NR | | none | NR | |
|  |  |  |  |  |  |  |  | patient 2 | |  |  | 33w+4 | 33 | C-section | maternal respiratory distress | | NR | |  |  |  |  | none |  |  |
|  |  |  |  |  |  |  |  | patient 3 | |  |  | 35w+3 | 34 | C-section | maternal respiratory distress | | ICU | |  |  |  |  | DM, hepatitis B |  |  |
|  |  |  |  |  |  |  |  | patient 4 | |  |  | 31w | 28 | C-section | maternal respiratory distress | | NR | |  |  |  |  | none |  |  |
|  |  |  |  |  |  |  |  | patient 5 | |  |  | 28w+5 | 37 | C-section | monochorionic diamniotic twins | | NR | |  |  |  |  | gestational DM |  |  |
|  |  |  |  |  |  |  |  | patient 6 | |  |  | 31w+5 | 32 | C-section | maternal respiratory distress | | NR | |  |  |  |  | None |  |  |
|  |  |  |  |  |  |  |  | patient 7 | |  |  | 37w+2 | 34 | vaginal (PROM) | NR | | NR | |  |  |  |  | gestational DM |  |  |
|  |  |  |  |  |  |  |  | patient 8 | |  |  | 33w | 25 | C-section | maternal respiratory distress | | NR | |  |  |  |  | none |  |  |
|  |  |  |  |  |  |  |  | patient 9 | |  |  | 26w | 32 | NR | NR | | NR | |  |  |  |  | chronic hypertension |  |  |
|  |  |  |  |  |  |  |  | patient 10 | |  |  | 34w+6 | 24 | vaginal | NR | | NR | |  |  |  |  | none |  |  |
|  |  |  |  |  |  |  |  | patient 11 | |  |  | 26w | 30 | NR | NR | | ICU | |  |  |  |  | none |  |  |
|  |  |  |  |  |  |  |  | patient 12 | |  |  | 25w+3 | 29 | NR | NR | | NR | |  |  |  |  | none |  |  |
| Andrikopoulou et al.,  (2020) | | NY | | Mar 13-April 19, 2020 | | 15 | | patient 1 | | RT-PCR (all positive) | | 37w+1 | NR | NR | NR | | ICU | | NR | | NR | | NR | discharged | |
|  |  |  |  |  |  |  |  | patient 2 | |  |  | 40w+5 | NR | NR |  |  | NR | |  |  |  |  | symptomatic hypoxia | readmission | |
|  |  |  |  |  |  |  |  | patient 3 | |  |  | 37w+5 | NR | NR |  |  | ICU | |  |  |  |  | acute kidney injury | discharged | |
|  |  |  |  |  |  |  |  | patient 4 | |  |  | 38w+2 | NR | NR |  |  | NR | |  |  |  |  | NR | discharged | |
|  |  |  |  |  |  |  |  | patient 5 | |  |  | 37w | NR | NR |  |  | ICU | |  |  |  |  | symptomatic hypoxia | discharged | |
|  |  |  |  |  |  |  |  | patient 6 | |  |  | 31w+5 | NR | NR |  |  | ICU | |  |  |  |  | NR | discharged | |
|  |  |  |  |  |  |  |  | patient 7 | |  |  | 32w+5 | NR | NR |  |  | NR | |  |  |  |  | NR | discharged | |
|  |  |  |  |  |  |  |  | patient 8 | |  |  | 30w+6 | NR | NR |  |  | ICU | |  |  |  |  | NR | readmission | |
|  |  |  |  |  |  |  |  | patient 9 | |  |  | 34w | NR | NR |  |  | ICU | |  |  |  |  | NR | readmission, COVID-19 | |
|  |  |  |  |  |  |  |  | patient 10 | |  |  | 30w+1 | NR | NR |  |  | NR | |  |  |  |  | NR | discharged | |
|  |  |  |  |  |  |  |  | patient 11 | |  |  | 25w+5 | NR | NR |  |  | ICU | |  |  |  |  | NR | discharged | |
|  |  |  |  |  |  |  |  | patient 12 | |  |  | 36w+3 | NR | NR |  |  | ICU | |  |  |  |  | NR | discharged | |
|  |  |  |  |  |  |  |  | patient 13 | |  |  | 32w+5 | NR | NR |  |  | NR | |  |  |  |  | symptomatic hypoxia | discharged | |
|  |  |  |  |  |  |  |  | patient 14 | |  |  | 31w+1 | NR | NR |  |  | ICU | |  |  |  |  | NR | discharged | |
|  |  |  |  |  |  |  |  | patient 15 | |  |  | 36w+3 | NR | NR |  |  | NR | |  |  |  |  | NR | NR | |

| Breslin et al., (2020) | New York | March 19, 2020 | 7 | patient 1 | PCR (all positive) | 37w | 38 | C-section | arrest of descent | ampicillin, gentamicin, acetaminophen, hydroxychloroquine | good | negative | type 2 DM, intrahepatic cholestasis of pregnancy | discharged |
| --- | --- | --- | --- | --- | --- | --- | --- | --- | --- | --- | --- | --- | --- | --- |
|  |  |  |  | patient 2 |  | 37w+5 | 33 | C- section | failed induction | Furosemide, hydroxychloroquine, azithromycin and ceftriaxone | good | negative | asthma, type 2 DM | hospitalized on postoperative day 5 |
|  |  |  |  | patient 3 |  | 35w+5 | 30 | NR | NR | ICU | NR | NR | none | discharged home on day 3 |
|  |  |  |  | patient 4 |  | 32w+5 | 32 | NR | NR | ICU | NR | NR | none | discharged home on day 3 |
|  |  |  |  | patient 5 |  | 26w+3 | 27 | NR | NR | NR | NR | NR | none | discharged |
|  |  |  |  | patient 6 |  | 28w | 38 | NR | NR | NR | NR | NR | none | discharged |
|  |  |  |  | patient 7 |  | 34w+6 | 39 | NR | NR | NR | NR | NR | asthma | discharged |

| Silverstein et al., (2020) | | New York | | NR | | 2 | | Patient 1 | | RT-PCR (all positive)  CT scan | | 36w+1 | | 17 | C-section | rapid respiratory decompensation | acetaminophen, azithromycin, vancomycin, hydroxychloroquine,  ventilation, tocilizumab | | ICU | | negative | | NR | | | discharged |
| --- | --- | --- | --- | --- | --- | --- | --- | --- | --- | --- | --- | --- | --- | --- | --- | --- | --- | --- | --- | --- | --- | --- | --- | --- | --- | --- |
|  |  |  |  |  |  |  |  | Patient 2 | |  |  | 34w | | 34 | C-section | O_2_ | azithromycin ceftriaxone, intubation followed by transfer to the ICU, tocilizumab | | ICU | | negative | | NR | | | discharged |
| Juusela et al., (2020) | | New Jersey | | March 2020 | | 2 | | Patient 1 | | RNA-PCR (both positive) | | 39w+2 | 45 | C-section | pre-eclampsia | | magnesium sulfate, methylprednisolone, hydroxychloroquine, norepinephrine, tocilizumab, ventilation | | ICU | | NR | | gestational DM, obesity, advanced maternal age | still in ICU | | |
|  |  |  |  |  |  |  |  | Patient 2 | |  |  | 33w+6 | 26 | C-section | acute heart failure, supraventricular tachycardia | | O_2_, ceftriaxone, azithromycin, metoprolol | | NR | | NR | | PCOS | hospitalized | | |
| Lucarelli et al., (2020) | | New Jersey | | March 31-May 20, 2020 | | 3 | | patient 1 | | RT-PCR (all positive) | | 28w | 38 | no delivery | NR | | ceftriaxone, azithromycin, hydroxychloroquine methylprednisolone betamethasone, ventilation, remdesivir | | ICU | | NR | | NR | discharged, still pregnant | | |
|  |  |  |  |  |  |  |  | patient 2 | |  |  | 29w+6 | 26 | no delivery | NR | | ceftriaxone, azithromycin, heparin, tylenol, hydroxychloroquine, furosemide , ventilation, beclomethasone prednisone, methylprednisolone | | ICU | | NR | | NR | discharged, still pregnant | | |
|  |  |  |  |  |  |  |  | patient 3 | |  |  | 23w+5 | 46 | no delivery | NR | | azithromycin, co-amoxiclav, hydroxychloroquine ceftriaxone, ventilation, methylprednisolone, tocilizumab, lovenox, betamethasone, continuous venovenous hemodialysis, red blood cells transfusion | | ICU | | NR | | acute hypoxic respiratory failure | discharged, still pregnant | | |
| Cooke et al., (2020) | | UK | | NR | | 2 | | patient 1 | | RT-PCR (both positive) | | 28w+4 | 39 | C-section | type 1 respiratory failure | | corticosteroids for fetal maturation, magnesium sulphate for fetal neuroprotection | | ICU, spontaneous bowel perforation | | negative | | BMI 42, type 2 DM | NR | | |
|  |  |  |  |  |  |  |  | patient 2 | |  |  | 28w+6 | 28 | C-section | respiratory failure | | corticosteroids for fetal maturation, magnesium sulphate | | good | | negative | | gestational DM |  |  |  |
| Govind et al., (2020) | | London UK | | March 7-March 22, 2020 | | 9 | | patient 1 | | RT-PCR (all positive) | | 39w | 33 | emergency C-section | sudden deterioration of respiratory function | | benzylpenicillin, gentamycin | | pyrexia and signs of pneumonia on day 6 | | positive | | gestational DM | NR | | |
|  |  |  |  |  |  |  |  | patient 2 | |  |  | 27w | 29 | emergency C-section | maternal respiratory function | | clarithromycin, cefuroxime, ventilation (10 days) | | ICU | | negative | | none |  |  |  |
|  |  |  |  |  |  |  |  | patient 3 | |  |  | 35w | 31 | emergency C-section | pathological cardiotocograph, not in labour | | NR | | poor feeding | | negative | | none |  |  |  |
|  |  |  |  |  |  |  |  | patient 4 | |  |  | 39w | 31 | C-section | breech | | NR | | talipes | | negative | | asthma |  |  |  |
|  |  |  |  |  |  |  |  | patient 5 | |  |  | 38w | 22 | vaginal | NR | | NR | | good | | negative | | none |  |  |  |
|  |  |  |  |  |  |  |  | patient 6 | |  |  | 37w | 39 | C-section | 3 previous CS | | NR | | good | | negative | | insulin dependent DM, hypertension |  |  |  |
|  |  |  |  |  |  |  |  | patient 7 | |  |  | 39w | 18 | C-section | Previous CS and maternal request | | NR | | good | | negative | | none |  |  |  |
|  |  |  |  |  |  |  |  | patient 8 | |  |  | 39w | 38 | C-section | 3 previous CS | | NR | | good | | negative | | none |  |  |  |
|  |  |  |  |  |  |  |  | patient 9 | |  |  | 39w | 34 | C-section | maternal request | | NR | | good | | negative | | none |  |  |  |
| Koumoutsea et al., (2020) | | Canada | | NR | | 2 | | Patient 1 | | PCR (both positive) | | 35w+3 | 40 | C-section | Repeat C-section | | piperacillin, tazobactam, filgrastim | | good | | negative | | gestational DM, neutropenia | discharged | | |
|  |  |  |  |  |  |  |  | Patient 2 | |  |  | 35w+2 | 23 | C-section | progressive coagulopathy and transaminitis | | fibrinigen, tranexamic acid | | NR | | NR | | asthma, obesity  (BMI 32) | discharged | | |
| Buonsenso et al., (2020) | | Italy | | March 19-March 24, 2020 | | 2 | | patient 1 | | RT-PCR (all positive) | | 37w+3 | 42 | C-section | NR | | NR | | good | | negative and discharged home and after 15 days the test was positive but the infant was asymptomatic | | good | NR | | |
|  |  |  |  | March 23-April 5, 2020 | |  |  | patient 2 | |  |  | 35w | 38 | C-section | fetal distress | | O_2_ for 48 hours | | good | | negative | | NR | positive RT-PCR test on placenta and umbilical cord, maternal breastmilk collected during the first 5 days of newborn life tested positive on 3 out of 5 samples | | |

| Buonsenso et al., (2020) | Rome Italy | NR | 4 | Patient 1 | RT-PCR LUS CT scan (all positive) | 24w | 31 | no delivery | NR | hydroxychloroquine, lopinavir/ritonavir, tolicizumab, | ICU | NR | NR | uncomplicated ongoing pregnancy |
| --- | --- | --- | --- | --- | --- | --- | --- | --- | --- | --- | --- | --- | --- | --- |
|  |  |  |  | Patient 2 |  | 38w | 42 | C-section at 40 weeks | NR | hydroxychloroquine, lopinavir/ritonavir | discharged in good condition | negative | NR | good |
|  |  |  |  | Patient 3 |  | 17w | 39 | no delivery | NR | hydroxychloroquine, lopinavir/ritonavir | NR | NR | NR | uncomplicated ongoing pregnancy |
|  |  |  |  | Patient 4 |  | 35w | 38 | C-section | fetal bradycardia | hydroxychloroquine, lopinavir/ritonavir | discharged in good condition | negative | NR | good |
| Hantoushzadeh et al., (2020) | Tehran Iran | mid-February to mid-March, 2020 | 9 | patient 1 | RT-PCR (all positive) | 30w+3 | 25-29 | vaginal | NR | oseltamivir, azithromycin, ceftazidime, linezolid, amantadine, meropenem, vancomycin, lopinavir/ritonavir | intrauterine fetal death | NR | NR | maternal death due to COVID-19 |
|  |  |  |  | patient 2 |  | 38w+3 | 25-29 | C-section | fetal distress | oseltamivir, azithromycin, ceftriaxone | NR | negative | NR | maternal death due to COVID-19 |
|  |  |  |  | patient 3 |  | 30w+5 | 40-44 | C-section | fetal distress | oseltamivir, vancomycin, meropenum | pneumonia on day 2, remains intubated in ICU | first negative and then tested positive at day 7 | NR | maternal death due to COVID-19 |
|  |  |  |  | patient 4 |  | 24w | 30-34 | NR | NR | hydroxychloroquine, oseltamivir, azithromycin, lopinavir/ritonavir and ceftriaxone. | NR | NR | NR | acute renal failure (serum creatinine 6 mg/dL), and within 24 hours suffered cardiopulmonary collapse and died after failed resuscitation. |
|  |  |  |  | patient 5 |  | 36w | 30-34 | C-section | impending cardiopulmonary collapse | ceftriaxone, oseltamivir, and lopinavir/ritonavir. | NR | NR | gestational DM | maternal death due to COVID-19 |
|  |  |  |  | patient 6 |  | dichorionic/diamniotic twin gestation at 24w | 35-39 | NR | NR | hydroxychloroquine, oseltamivir, and lopinavir/ritonavir ceftriaxone, azithromycin, vancomycin, and meropenum | intrauterine fetal death | NR | NR | maternal death |
|  |  |  |  | patient 7 |  | 28w twin gestation | 45-49 | C-section | maternal hypoxemia | hydroxychloroquine, oseltamivir, and lopinavir/ritonavir; 24 hours later empiric intravenous immunoglobulin | premature twins | negative | NR | maternal death |
|  |  |  |  | patient 8 |  | 33w+5 | 35-39 | C-section | fetal distress | hydroxychloroquine, oseltamivir, lopinavir/ritonavir, meropenum, vancomycin, azithromycin, levofloxacin, COVID-19 convalescent plasma transfusion | NR | NR | NR | on ventilator support |
|  |  |  |  | patient 9 |  | 36w | 35-39 | C-section | intrauterine fetal death | fresh frozen plasma, second-generation cephalosporin | NR | NR | Gestational diabetes mellitus | slow recovery and still in hospital |
| Khan et al., (2020) | China | Jan 28-Mar 1, 2020 | 3 | Patient 1 | qRT-PCR (a;l positive) | 34w+6 | 28 | vaginal | NR | azithromycin, oseltamivir, chinese medicine | good | negative | NR | NR |
|  |  |  |  | Patient 2 |  | 39w+1 | 33 | vaginal | NR | antibiotics, antivirals, O_2_ | good | negative | NR | NR |
|  |  |  |  | Patient 3 |  | 38w+2 | 27 | vaginal | NR | antibiotics,antivirals, Chinese medicine, O_2_ | good | negative | NR | NR |
| Doria et al., (2020) | Portugal | March 25-April 15, 2020 | 12 | patient 1 | RT-PCR (all positive) | 35w | 22 | NR | NR | NR | FGR | NR | none | NR |
|  |  |  |  | patient 2 |  | 30w | 41 | NR | NR |  | PROM | NR | ulcerative colitis, psoriasis (chronic corticosteroid therapy) |  |
|  |  |  |  | patient 3 |  | 37w | 36 | C-section | NR |  | FGR | negative | Severe Scoliosis, Behçet Syndrome |  |
|  |  |  |  | patient 4 |  | 39w | 38 | C-section | NR |  | FGR | negative | gestational DM |  |
|  |  |  |  | patient 5 |  | 41w | 27 | C-section | NR |  | NR | negative | none |  |
|  |  |  |  | patient 6 |  | 37w | 32 | vaginal | NR |  | NR | negative | gestational hypertension |  |
|  |  |  |  | patient 7 |  | 40w | 33 | C-section | NR |  | FGR | negative | severe myopia |  |
|  |  |  |  | patient 8 |  | 40w | 34 | vaginal delivery | NR |  | FGR | negative | none |  |
|  |  |  |  | patient 9 |  | 37w | 35 | vaginal delivery | NR |  | FGR | negative | asthma, Raynaud syndrome |  |
|  |  |  |  | patient 10 |  | 39w | 20 | vaginal | NR |  | NR | NR | none |  |
|  |  |  |  | patient 11 |  | 38w | 29 | C-section | NR |  | FGR | negative | chronic hypertension |  |
|  |  |  |  | patient 12 |  | 37w | 29 | C-section | dichorionic diamniotic twins |  | fetal growth discordance | negative/negative | None |  |
| Yassa et al., (2020) | Istanbul Turkey | NR | 8 | patient 1 | rRT-PCR (all positive) | 39w | 32 | C-section | PROM | hydroxychloroquine | good | negative | NR | good |
|  |  |  |  | patient 2 |  | 27w | 32 | NR | NR | hydroxychloroquine, azithromycin, oseltamivir, | NR | NR |  | discharged, uncomplicated ongoing pregnancy |
|  |  |  |  | patient 3 |  | 20w | 33 | NR | NR | ritonavir/lopinavir, hydroxychloroquine, azithromycin, meropenem, favipiravir, | ICU | NR |  | stable, uncomplicated ongoing pregnancy |
|  |  |  |  | patient 4 |  | 9w | 19 | NR | NR | ritonavir/lopinavir | NR | NR |  | good, uncomplicated ongoing pregnancy |
|  |  |  |  | patient 5 |  | 17w | 41 | NR | NR | hydroxychloroquine, azithromycin, ritonavir/lopinavir | NR | NR |  | good, uncomplicated ongoing pregnancy |
|  |  |  |  | patient 6 |  | 7w | 40 | NR | NR | hydroxychloroquine, azithromycin | NR | NR |  | abortion, asymptomatic |
|  |  |  |  | patient 7 |  | 10w | 23 | NR | NR | ritonavir/lopinavir, azithromycin | NR | NR |  | good,  uncomplicated ongoing pregnancy |
|  |  |  |  | patient 8 |  | 38w | 40 | C-section | maternal request | favipiravir, hydroxychloroquine, azithromycin | NR | NR |  | good |
| Huang W et al., (2020) | China | Jan 24-Feb 19, 2020 | 8 | patient 1 | RT-PVR (all positive) | 30w | 29 | ongoing pregnancy | NR | NR | good | none | mild anemia | good |
|  |  |  |  | patient 2 |  | 34w | 27 | ongoing pregnancy | NR |  | good | none | none | good |
|  |  |  |  | patient 3 |  | 39w+3 | 28 | C- section | NR |  | good | negative | none | good |
|  |  |  |  | patient 4 |  | 38w | 33 | C- section | NR |  | good | negative | none | good |
|  |  |  |  | patient 5 |  | 37w+4 | 29 | emergency C- section | NR |  | good | negative | mild anemia | good |
|  |  |  |  | patient 6 |  | 31w+2 | 29 | vaginal | NR |  | twins, ICU (both severe neonatal  asphyxia, one neonatal death) | both negative | mild anemia, eclampsia, PROM | good |
|  |  |  |  | patient 7 |  | 35w+2 | 32 | emergency C- section | NR |  | fetal death | none | septic shock, SICM, ARDS, MODS | good |
|  |  |  |  | patient 8 |  | 28w+1 | 32 | emergency C- section | NR |  | severe neonatal asphyxia, ICU | negative | moderate anemia | good |

*COVID-19: coronavirus disease 2019, w: weeks, NR: not reported, C-section: caesarian section, qRT-PCR :* *quantitative reverse transcription polymerase chain reaction, CT: computed tomography, ICU : intensive care unit, IUFD: intrauterine fetal death, O_2_: oxygen,* *PROM*: *premature rupture of membranes, DM: diabetes mellitus, FGR: fetal growth restriction, PCOS: polycystic ovary syndrome, MODS: multiple organ dysfunction syndrome, ARDS: acute respiratory distress syndrome , SICM: sepsis-induced cardiomyopathy, ECMO: extracorporeal membrane oxygenation*

Table 3: Cohort studies examining pregnancies affected by COVID-19

| **First author (year)** | **Region, country** | **Study period** | **Sample size** | **Gestational age** | **Test for COVID-19** | **Maternal age (years)** | **Mode of delivery** | **Indication for C-section** | **Treatment received** | **Newborn health** | **SARS-CoV-2 status of the newborn** | **Maternal comorbidities** | **Maternal health at the end of study** | **Special remarks** |
| --- | --- | --- | --- | --- | --- | --- | --- | --- | --- | --- | --- | --- | --- | --- |
| Liu et al.,  (2020) | Wuhan China | Jan 20-Feb 10 | 15 | 12-38w | qRT-PCR  (all positive) | 23-40 | 10 (66.6%)  C-section,  1 (6.67%) vaginal,  4 (26.67%)  still pregnant | NR | O_2_, antiviral,  antibiotic, O2 | NR | NR | thalassemia (1), mitral and tricuspid valve replacement (1), complete placenta previa (1) | good | NR |
| Chen et al.,  (2020) | Wuhan China | Jan 30-Feb 23 2020 | 17 | NR | RT-PCR  (all positive) | NR | all C-section | NR | NR | 3/17 premature | negative | gestational hypertension (1), gestational DM (2) | 3 still recovering | 14/17 continuous epidural anesthesia , 12/14 significant hypotension |
| Wu et al.,  (2020) | Wuhan | December 31 2019 -March 7 2020 | 23 | 20>28 weeks  3 <12weeks | RT-PCR  (all positive) | 21-37 | C-section  18 (78.26%) ,  vaginal  2 (8.7%),  terminated voluntarily  3 (13.04%) | fetal distress | NR | good | 4 negative RT-PCR,  17 negative clinically | hypothyroidism (2), hepatitis B (2), gestational hypertension (4) | good | NR |
| Liu et al.,  (2020) | China | January 27-February 27 | 59 (16 laboratory confirmed, 25 diagnosed clinically) | 22w - 40w+5 | RT-PCR  (41 positive) | 22-42  (mean age 30) | NR | NR | antiviral | NR | NR | 4 gestational DM,  3 gestational hypertension,  1 hepatitis B | NR | NR |
| Yan et al., (2020) | China | Jan 20- March 24 | 116 pregnant from 25 hospitals | 38w  (median) | qRT-PCR (all positive) | 24-41  (mean 30.8) | 99 delivered (85/99 C-section,  14/99 vaginal) | 33/85 pneumonia,  16/85 previous C-section,  9/85 fetal distress,  5/85 failure to progress | antiviral,,antibiotic, corticosteroid | 1 neonatal death | 86/100 negative | NR | 76/116 discharged , 1/116 ECMO, 2 invasive mechanical ventilation, 1/116 plasmapheresis , 40/116 still in hospital | NR |
| Liu W et al., (2020) | China | Jan 31- Feb 29 | 19 neonates | 38+6w | RT-PCR | 27-34 | C-section  (18/19)  vaginal  (1/19) | NR | 6/19 antivirals before delivery | NR | negative | none | NR | NR |
| Wu et al., (2020) | China | Jan 31- March 9 | 13 pregnancies | 5w-38w | RT-PCR  (all positive) | 26-40 | 3rd trimester (5/13),  vaginal (1/5),  C-section (4 /5) | fetal distress (1),  medical indication (3) | O_2_ support to all, 8/13 antiviral and antibacterial treatment, 3/13 corticosteroids | 2 premature neonates with pneumonia | negative | NR | NR | all 13 vaginal secretions samples negative, only 1 stool sample positive |
| Qiancheng et al., (2020) | China | Jan 15-March 15 | 82 (28 pregnant, 54 non-pregnant women) | 38w (median) , three in 1st trimester, one in 2nd trimester, 24 in 3rd trimester | RT-PCR (all 28 pregnant women positive) | 18-41  (median 30) | 23 live births (included two twins),  C-section (17, 60.7%), vaginal (5, 17.9%) | NR | 21 (75%) of pregnant antiviral  (20, 71.4% ribavirin alone), all non-pregnant antivirals  19 (35.2%) ribavirin,  11 (20.4%) umifenovir, 17 (31.5%), ribavirin and umifenovir,  7 (13.0%) interferon-alpha inhalation, ribavirin and umifenovir,  24, 85.7% vs. 47, 87% in both groups prophylactic antibiotics, non-pregnant women more corticosteroids (21, 38.9% vs. 4, 14.3%) and immunoglobulins (19, 35.2% vs. 3, 10.7%) than pregnant | good | negative | among pregnant women, 1 (3.6%) had probable gestational hypertension, 2 (7.1%) had probable gestational DM, 2 (7.1%) had chronic hepatitis B, 1 (3.6%) had hypothyroidism; among non-pregnant women, 4 (7.4%) had DM, 2 (3.7%) had chronic hepatitis B, 1 (1.9%) had hypothyroidism | no fatal case | no association between pregnancy and severity of disease, virus clearance time and length of hospital stay |
| Yang et al., (2020) | China | January 20 - March 19 | 27 | 4 in 1^st^ trimester and 23 pregnant women 30 w-40 w | RT-PCR (all positive) | 22-39w | 4 abortion, 18/27 C-section,  5/27 vaginal | NR | O_2_, antibiotics (all), antivirals– arbidol, ribavirin (19) corticosteroids (1 patient with severe pneumonia) | discharged | 23/24 had negative RT-PCR SARS-CoV-2 testing of throat swab --among these, one preterm newborn, born to mother with PPROM, showed elevated IgG and Ig M level for SARS-CoV-2 2-hours after the birth, but negative for repeated RT-PCR testing (twice) of the swab samples | hepatitis B (2), schistosomiasis (1), gestational DM (3), blood coagulation dysfunction (3),  gestational hypertension (2), hypothyroidism (2), severe pre-eclampsia (1), hypoproteinemia (1) | discharged | NR |
| Wang et al., (2020) | Wuhan, China | December 8, 2019-April 1 | 72 women (30 pregnant and 42 non-pregnant) | 37.8  (median) | RT-PCR CT scan (all 30 positive) | 30w (median) | 23 C-section,  7 vaginally | NR | NR | NR | NR | obesity, hypothyroidism. hypertension, DM, intrahepatic cholestasis of pregnancy | pregnant patients fully recovered | pregnant patients admitted to hospital earlier (0.25 vs 11.00 days), presented milder symptoms, had higher rate of asymptomatic infection (26.7% vs 0%), shorter length of hospital stay (14.5 vs 17.0 days) compared with non-pregnant |
| Zeng QL et al., (2020) | Henan and Shaanxi Provinces, China | January 20-March 26 | 149 (3 pregnant) | 30w - 37w | RT-PCR (all 3 positive) | NR | C-section | fetal heart rate instability and possible fetal respiratory distress | ARDS, ventilation, ECMO (1) | pneumonia, ICU (1) | negative (2) | NR | death (1) due to COVID-19 | NR |
| Zeng et al., (2020) | Wuhan, China | February 16 | 16 pregnancies | 37+5w | RT-PCR (all positive) | 31 ± 3.84 | C-section (12) vaginal (4) | PROM (3), preterm birth (3), FGR (1), fetal macrosomia (1) | antibiotics (16), antivirals (16), glucocorticoids (6), O_2_ (7) | transferred to children’s hospital for isolation (12),  preterm (3) | all negative | cardiac disease (2), hypothyroidism (2), polyhydramnios (1), thalassemia (1) | NR | NR |
| Liao et al., (2020) | Wuhan, China | January 20-March 2 | 63 | NR |  |  |  |  |  |  |  |  |  |  |
|  |  |  | 10 pregnant with COVID-19 |  | RT-PCR  (10 positive) | 38.5 ± 1.43 | 31.90 ± 3.35 | vaginal | NR | NR | discharged (3), transferred to neonatal isolation (7) | 7 negative | postpartum hemorrhage, premature delivery (1) | NR |
|  |  |  | 53 pregnant without  COVID-19 |  | NR | 38.57 ± 1.99 | 29.52 ± 3.31 | vaginal | NR | NR | NR | NR | postpartum hemorrhage, premature delivery (5) | NR |
| Chen Lian et al., (2020) | Wuhan, China | December 8 2019-March 20, 2020 | 118 pregnant women with COVID-19 | NR | PCR (all positive) | 28-34 (median 31) | 3 spontaneous abortions, 2 ectopic pregnancies, and  4 induced abortions | 68/118 patients (58%) delivered during the study period, (0.56% of all deliveries in Wuhan), and had 70 births (2 sets of twins).  63/68 (93%) C-sections | non-invasive mechanical ventilation | 14 premature deliveries (21%); 8 induced (7 owing to concern about COVID-19) | negative | NR | 109/116 (94%) discharged, including all women with severe or critical disease | NR |
| Shanes et al., (2020) | Chicago | March 18-May 5 | 16 pregnancies | 16w-40w | RT-PCR (all positive) | 26-41 | NR | NR | NR | 1 intrauterine death at 16w, 1 inpatient, the rest discharged | 14/16 negative , | 1 patient was hypertensive , 1 had cholestasis of pregnancy and gestational DM,  1 IUFD | NR | increasd rates of maternal vascular malperfursion |
| Miller et al., (2020) | Chicago, USA | April 8-April 27 | 635 | NR | PCR (46 positive) | NR | NR | NR | NR | NR | NR | NR | NR | NR |
| Breslin et al., (2020) | New-York | March 13- March 27 | 43 pregnancies | 37w  (median) | RT-PCR (all positive) | 20-39 | 8 /18 C-section 10/18 vaginal delivery | fetal distress (3), previous C-section (2), arrest of descent (1) arrest of dilation (1) failed labor induction (1) | hydroxychloroquine, ceftriaxone (1), ceftriaxone, azithromycin (1), hydroxychloroquine (1) | discharged | 18 neonates negative | 8 asthma, 3 DM, 3 chronic hypertension | 1 still inpatient | 2 patients in ICU (1 with renal insufficiency) |
| Penfield et al., (2020) | New York | NR | 11 pregnancies | 26w+5-41w+3 | RT-PCR (all positive) | 22-40 | 4/11 C-section 7/11 vaginal | NR | NR | NR | negative | NR | NR | 3/11 placental or membrane swabs positive |
| Fox et al., (2020) | NY | March 20-April 30 | 92 pregnancies | NR | PCR (all positive) | 31.4  (median) | 21 deliveries | NR | NR | NR | NR | NR | No death | NR |
| Blitz et al., (2020) | NY | March 1-May 6 | 13 admitted to ICU | 33.3±5.3 w  (mean) | PCR (all positive) | 33.8±5.2 | 6 urgent  C-sections,  1 vaginal | acute respiratory decompensation, cord prolapse | anticoagulation, hydroxychloroquine, antibiotics, remdesivir, interleukin-6-receptor inhibitor, convalescent plasma therapy | 4 preterm births | negative | asthma (2), obstructive sleep apnea (1), DM (1), gestational DM (1), gestational hypertension,  pre-eclampsia (3) | 2 deaths | NR |
| Khoury et al., (2020) | NY | March 13- April 12 | 241 | NR | RT-PCR (all positive) | 32 | vaginal (141), C-section (100) | fetal deceleration23), failed induction (11), worsening respiratory distress (10), arrest (5), arrest of descent (5), malpresentation (5), previous C-section (31), other (10) | NR | 30% resuscitated, 25.7% ICU, majority (62.4%) hospitalized < 2 days, commonest newborn complications due to prematurity or low birth weight | of 236 liveborn neonates with documented SARS-CoV-2 test results, 230 (97.5%) tested negative for SARS-CoV-2 infection within 24–96 hours of life | admission to ICU (17, 7.1%),  intubation during  delivery  (9, 3.7%) | NR | statistically significant linear trend of COVID-19 severity with risk of C-section |
| London et al., (2020) | Brooklyn, New York | March 15-April 15 | 81 | PCR (68 positive) |  |  |  |  |  |  |  |  |  |  |
|  |  |  | symptomatic (46) |  | NR | 30 | C-section (16) | pre-eclampsia (2), preterm birth <37 weeks (9), preterm birth <34 weeks (3) | respiratory support (12), hydroxychloroquine and azithromycin (16) | NR | negative | postpartum hemorrhage (1) | no deaths | NR |
|  |  |  | asymptomatic (22) |  | NR | 30,5 | C-section (6) | pre-eclampsia (1) | None | NR | negative | postpartum hemorrhage (1) | no deaths | NR |
| Emeruwa et al., (2020) | NY | March 22-April 21 | 396 | 39w  (38.1-39.7) | PCR (71 positive) | 31 (27-35) | NR | NR | NR | NR | NR | NR | NR | SARS-CoV-2 transmission among pregnant women in New York City was associated with neighborhood- and building-level markers of large household membership, household crowding, and low socioeconomic status |
| Lokken et al., (2020) | Washington, USA | January 21-April 17 | 46 pregnancies | NR | PCR (all positive) | 29 | 8 vaginal (5)  C-section (3) | previous C-section, fetal status, DM, respiratory compromise, arrest, malpresentation, COVID-19, cholestasis, history of shoulder dystocia, fetal macrosomia | hydroxychloroquine and remdesivir (1), remdesivir alone (2), azithromycin (2), ICU (1) | 7 living, 1 stillbirth | negative | type 2 DM (3), asthma (4), hypothyroidism (3), hypertension (2), Crohn’s disease (1), heart valve repair (1), thyroidectomy (1), seizures (2) | NR | NR |
| Goldfarb et al., (2020) | USA | March 6-May 4 | 192 (65 Hispanic 127 non-Hispanic) 136 tested for COVID-19 (39 Hispanic 22 non-Hispanic positive) | pregnant or 2 weeks post patrum | RT-PCR (61 positive) | symptomatic (Hispanic mean 30, non-Hispanic mean 34) confirmed cases (Hispanic mean 29 non-Hispanic 35) | NR | NR | NR | NR | NR | obesity, DM, asthma | no deaths | NR |
| Qadri et al., (2020) | Michigan | March 26-April 10 | 16 pregnancies | 20w-40w+3 | RT-PCR (all positive) | 20-40 | spontaneous vaginal (8/16) C-section  (4/16) | 2 /16 C-section due to obstetrical indication, 1/16 repeat C-section , 1/16 elective C-section | 2/16 received O2 by nasal cannula , azithromycin, hydroxychloroquine, remdesivir and steroids | good | negative | NR | discharged | NR |
| Pierce-Williams et al., (2020) | Philadelphia USA | March 5 - April 20 | 64 | 30w+-6  (mean) | PCR (all positive) | mean age 33 | 44 patients severe disease; 15 delivered (7 vaginal and 8 C-sections) 29 didn't deliver | NR | hydroxychloroquine (8), remdesivir (10), antibiotics (8) | 64% ICU | 1/33 neonates diagnosed with COVID-19 48 hours after delivery | pre-eclampsia or gestational hypertension in 2 women | NR | NR |
| Campbell et al., (2020) | Connecticut | April 2-April 29 | 30/770 positive | <37 w (0) | PCR (30 positive) | <30 (14), 30-34 (10), ≥35 (6) | C-section (10) | NR | NR | NR | 0 | NR | NR | prevalence of positive test among asymptomatic patients 2.9% (22/756) |
|  |  |  | 740/770 negative | <37 w (62) |  | <30 (199), 30-34 (310), ≥35 (231) | C-section (275) | NR | NR | NR | NR | NR | NR | NR |
| Ferrazzi et al., (2020) | Northern Italy | March 1 -March 20 | 42 pregnancies | NR | RT-PCR  (all positive) | 21-44(mean maternal age 32.9) | 24/42 vaginal 18/42 C-section - | (8) indication unrelated to COVID-19, (10) worsening dyspnoea or other COVID-19 symptoms | NR | NR | 1 newborn from a COVID-19 mother delivered vaginally at term had equivocal test after delivery and positive test 3 days later | 6/42 gestational DM | NR | NR |
| Savasi et al., (2020) | Italy | February 23-March 28 | 77 pregnancies | 261 days | RT-PCR  (all positive) | 32 | 57/77 delivered; vaginal (34),  C-section (22) | NR | antibiotic (27), antiviral (25), hydroxychloroquine (19), O_2_ (20) | ICU (9) | positive (4) | obesity, cardiovascular, autoimmune, endocrine, metabolic diseases (24) | 20 still pregnant at discharge | NR |
| Gagliardi et al., (2020) | North Tuscany and Liguria, Italy | up to April 19, 2020 | 533 pregnancies | NR | PCR  (3 positive) | NR | NR | NR | NR | no problems | NR | NR | NR | estimated 83% (51-94) of infections unreported |
| Patane et al., (2020) | Bergamo, Italy | March 5-April 21 | 22 (2 neonates positive) | NR | RT-PCR  (all positive) | NR | vaginal (1)  C-section (1) | non- reassuring fetal status (1) | NR | good (1) ICU (1) | positive (1) positive after 7-days (1) | NR | discharged on day 10 (1),  on day 20 (1) | NR |
| Pereira et al., (2020) | Madrid, Spain | March 14-April 14 | 60 | 1st trimester (1-12 weeks) 10, 2nd trimester (13-26 weeks) 16, 3rd trimester (27-41 weeks) 34 | RT-PCR (all positive) | <30 y (11), 30-34 (24), 35-40 y (22), >40 y (3) | spontaneous vaginal (14), instrumental (4), C-section (5), premature delivery (2) | FGR, pre-eclampsia | hydroxychloroquine (10), hydroxychloroquine + lopinavir + ritonavir (3), hydroxychloroquine + darunavir + ritonavir (3), hydroxychloroquine + darunavir + ritonavir + tocilizumab (2), hydroxychloroquine + darunavir + cobicistat (3) | good | all negative | NR | NR | NR |
| Martinez-Perez et al., (2020) | Spain | March 12-April 6 | 82 | NR | RT-PCR  (all positive) |  |  |  |  |  |  |  |  |  |
|  |  |  | asymptomatic/mild vaginal symptoms (41) | 38w+1 |  | 35 | vaginal | NR | NR | ICU (8) | none | hypothyroidism (3), epilepsy (1),Subek Muscular dystrophy (1), myopathy (1), heterozygous factor V mutation (1), psychiatric disorders(3), autoimmune disease (1), hyperprolactinemia (1), gastritis (1), vitiligo (1), chronic hepatitis C (1), gestational DM (1), pre-eclampsia (3) asthma (3) | clinical deterioration (2) | NR |
| Mendoza et al., (2020) | Barcelona, Spain | March 13-April 10 | 42 pregnancies;  severe COVID-19  (8), non severe (34) | 31.6w | RT-PCR  (all positive) | 32 | C-section during ICU stay (4) | HELLP syndrome in one case and worsening COVID-19 symptoms in the other 3 cases | NR | NR | NR | NR | 1 DM | NR |
| Knight et al., (2020) | UK | March 1-April 14 | 427 | 34w  (median) | RT-PCR  (all positive) | 4 <20 ,  248 20-34 175 >35 | C-section (156) vaginal (106) | 42 women (27% of C-section) due to maternal compromise, 37 (24%) due to fetal compromise, 30 (19%) due to failure to progress, 25 (16%) due to other obstetric reasons, 16 (10%) due to previous C-section,  6 (4%) at maternal request | 9 (2%) antivirals (oseltamivir, lopinavir/ritonavir, remdesivir)  64 (15%) corticosteroids for fetal lung maturation | 5 babies died; 3 stillborn, 2 died in neonatal period, 3 deaths due to obstetric conditions, 67/265 (25%) liveborn infants in ICU,  1 infant with neonatal encephalopathy | 12 infants tested positive | asthma ,hypertension , DM, cardiac disease | 5 died due to COVID-19,  397 discharged well, 25 still in hospital | NR |
| Khalil et al., (2020) | UK | April 20 | 129 women tested on admission; 9 (7.0%)  positive,  8/9 (88.9%) asymptomatic | NR | RT-PCR (9 positive) | 34  (median) | NR | NR | NR | all babies good and discharged | NR | asthma (1) | discharged | 7.0% of pregnant 72 women attending hospital for delivery were positive for SARS-CoV-2 and 8 of the 9 positive women were asymptomatic. |
| Sentilhes et al., (2020) | Strasbourg, France | March 1-April 3 | 54 (21 deliveries) | 37.4 ± 4.7w | qRT-PCR  (38 positive,  16 suspected) | 30.6 ± 6.2 | C-section (9) vaginal (12) | COVID-19 | NR | intubation in labor ward (3), ICU (3), CPAP in first 24 h (3), blood products transfusion (1), phototherapy (1) | all negative | chronic hypertension (1), asthma (5), other chronic disease (4), previous C-section (9), history of postpartum hemorrhage (2), gestational DM (4), gestational hypertensive disorders (2) | NR | NR |
| Kayem et al., (2020) | France | March 1-April 14 | 617 | NR | RT-PCR CT scan (all positive) | NR | 181 women, mode of delivery not mentioned | NR | NR | 1 neonatal death in critical group due to prematurity | 2 neonates SARS-CoV-2 RT-PCR positive | NR | disease severity associated with age > 35, obesity, DM, pre-eclampsia | 1 woman with COVID-19 died |
| Collin *et al*., (2020) | Sweden | March 19-April 20 | 52 women (11 pregnancies, 2 postpartum) | 13w-40w | PCR (all 13 positive) | 20-35 | NR | NR | all pregnant or postpartum women required ICU;  7/13 invasive mechanical ventilation; all discharged from ICU (median stay 6 six days, range <1-21 days)-  among 40 non-pregnant women, 29 required invasive mechanical ventilation | NR | NR | NR | NR | risk of requiring ICU may higher in pregnant women with laboratory-confirmed SARSCoV-2 in Sweden, compared to non-pregnant women of similar age |

*COVID-19: coronavirus disease 2019, w: weeks, NR: not reported, C-section: caesarian section, qRT-PCR :* *quantitative reverse transcription polymerase chain reaction, CT: computed tomography, ICU : intensive care unit, IUFD: intrauterine fetal death, O_2_: oxygen,* *PROM*: *premature rupture of membranes, DM: diabetes mellitus, FGR: fetal growth restriction, PCOS: polycystic ovary syndrome*

Table 4: Case-control studies examining pregnancies affected by COVID-19

| **First author (year)** | **Region, country** | **Study period** | **Sample size** | **Gestational age** | **Maternal age (years)** | **Mode of delivery** | **Treatment received** | **Outcomes studied** | **Newborn health** | **SARS-CoV-2 status of the newborn** | **Maternal comorbidities** | **Maternal health at the end of study** | **Special remarks** |
| --- | --- | --- | --- | --- | --- | --- | --- | --- | --- | --- | --- | --- | --- |
| Tekbali et al., (2020) | NY | March 2-March 29 | 21,980  (3,064 pregnant or post-patrum,  18,916 non- pregnant) | NR | NR | NR | NR | NR | pregnant and non-pregnant severe acute respiratory syndrome coronavirus 2 and coronavirus disease 2019 hospital admissions | NR | NR | NR | SARS-CoV-2 status for pregnant and postpartum patients increased from 0.14% in week 1 to 5.65% of all hospital admissions in week 4  SARS-CoV-2 status for non-pregnant patients increased from 1.21% in week 1 to 56.79% of all hospital admissions in week 4 |
| Li et al., (2020) | Wuhan | Jan 24- Feb 29 2020 | 16 positive,  18 suspected | 33w^+6^-40w^+4^ | 26-37 | 14/16 16/18  C-section | 16 antibiotics,  4 antiviral  (positive group)  18 antibiotics  (suspected group) | comparison of pregnant with COVID-19 with pregnant without COVID-19 | no severe asphyxia or death | negative | gestational DM (3) PROM (1), gestational hypertension (3), hypothyroidism (1),  pre-eclampsia (1),  sinus tachycardia (1) | 8 discharged  8 transferred  *vs.*  18 discharged |  |
|  |  |  |  |  |  | C-section control group A 57/121,  control group B 44/121 |  |  |  |  |  |  |  |
|  |  |  | Control group A:121 women  B: 121 women |  |  |  |  |  |  |  |  |  |  |

*COVID-19: coronavirus disease 2019, w: weeks, NR: not reported, C-section: caesarian section, PROM*: *premature rupture of membranes, DM: diabetes mellitus*
